# Microalgae production in human urine: Fundamentals, opportunities, and perspectives

**DOI:** 10.3389/fmicb.2022.1067782

**Published:** 2022-11-18

**Authors:** Yewen Tao, Zhipeng Liu, Junjian Zheng, Jieqin Zhou, Di He, Jinxing Ma

**Affiliations:** ^1^Key Laboratory for City Cluster Environmental Safety and Green Development of the Ministry of Education, School of Ecology, Environment and Resources, Guangdong University of Technology, Guangzhou, China; ^2^School of Environmental Science and Engineering, Guangdong University of Technology, Guangzhou, China; ^3^College of Life and Environmental Sciences, Guilin University of Electronic Technology, Guilin, China

**Keywords:** microalgae, urine, photobioreactor, biomass, ammonia, resource recovery

## Abstract

The biological treatment of source-separated human urine to produce biofuel, nutraceutical, and high-value chemicals is getting increasing attention. Especially, photoautotrophic microalgae can use human urine as media to achieve environmentally and economically viable large-scale cultivation. This review presents a comprehensive overview of the up-to-date advancements in microalgae cultivation employing urine in photobioreactors (PBRs). The standard matrices describing algal growth and nutrient removal/recovery have been summarized to provide a platform for fair comparison among different studies. Specific consideration has been given to the critical operating factors to understand how the PBRs should be maintained to achieve high efficiencies. Finally, we discuss the perspectives that emphasize the impacts of co-existing bacteria, contamination by human metabolites, and genetic engineering on the practical microalgal biomass production in urine.

## Introduction

Urine contains a large amount of nitrogen, phosphorus, potassium and other nutrients. While contributing to only 1% of the total volume of sewage, human urine accounts for 80% and 40%–50% of the total nitrogen and phosphate loads, respectively (Wilsenach et al., 2007). Conventional management of urine in wastewater treatment is not only energy-intensive (e.g., at an energy cost of 45 MJ kg-N^−1^ and 49 MJ kg-P^−1^) but also leads to the loss of nitrogen and phosphorus resources through waste discharge (Maurer et al., 2003; Liu Z. et al., 2008). As a result, a separate collection of urine has presented an exciting alternative to sewage management (Nazari et al., 2020). For example, no-mix technology can produce source-separated urine that only contains water, urea and inorganic salts (e.g., Ca^2+^ and Mg^2+^; Wilsenach and van Loosdrecht, 2006), which has been considered a promising but unexploited stock for N and P fertilizers for agriculture (Larsen et al., 2021). Therefore, we envision that source separation and utilization of urine can not only improve the sustainability of sewage management but also add the potential to achieve the minimum environmental impacts over a fertilizer life cycle.

Conventional treatment processes, including stripping, physical adsorption, and chemical precipitation, have been deployed to manage urine and separate nutrients; however, they suffer from the process limitations such as high energy consumption, significant chemical dose, and ammonia loss (Zhang et al., 2018). Alternatively, the biological treatment of urine, mainly to produce biofuel, nutraceutical and high-value chemicals is getting increasing attention (Soares et al., 2013). Microalgae are photoautotrophic microorganisms that take up and accumulate nutrients using light as an energy source and carbon dioxide as an inorganic carbon source (Tuantet, 2015). The cultivation of microalgae requires nutrients, primarily N and P. In addition, the urine also comprises trace elements (e.g., B, Cu, Zn, Mo, Fe, Co and Mn) that are necessary for algal growth. Furthermore, human urine typically contains no hazardous chemical compounds or heavy metals (Rodushkin and Ödman, 2001; Gòdia et al., 2002). To this end, using source-separated urine as media has been a practice for environmentally and economically viable large-scale microalgae cultivation for biofuel production.

**Figure 1:**
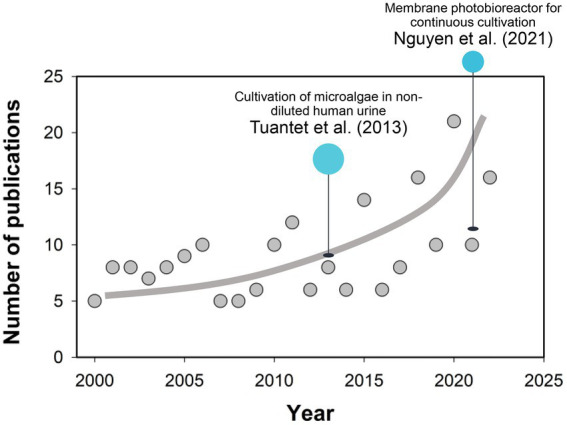
Survey of the publications (Elsevier Scopus) in topics related to “(Micro)algae” and “Urine” from 2000 to 2022 (Accessed date: October 29, 2022). Line serves to guide the eye.

According to the literature available, Tuantet et al. (2013) pioneered the cultivation of microalgae in non-diluted human urine, in which fresh and synthetic urine was first demonstrated to support the rapid growth of *Chlorella sorokiniana*, highlighting the significance of economically large-scale microalgae production in human urine (Tuantet et al., 2013). Jaatinen et al. (2016) also successfully cultivated *Chlorella vulgaris* in 100-times diluted urine at the highest biomass density of 0.60 g L^−1^. Following attempts have been carried out employing various species such as *Spirulina* and *Scenedesmus acuminatus*. Moreover, microalgae production in human urine also achieves the removal and recovery of nutrients (i.e., N and P). In the study by Chang et al. (2013), 97% of ammonium nitrogen, 96.5% of the total phosphorus (TP) and 85%–98% of urea in diluted urine could be removed by microalgae, the practice of which has closed the gap between waste management and sustainable resource exploitation (Behera et al., 2020). With regard to the challenges in biomass separation from the dilute media, a membrane photobioreactor (MPBR) has been proposed and deployed for the continuous cultivation of microalgae (Nguyen et al., 2021). An unofficial Scopus search of the literature on “(Micro)algae” and “Urine” was carried out for the timeline from 2000 to 2022 (Figure 1), with the results demonstrating a quick increase in the publication number in the past decade. While recent progress in microalgae breeding and reactor design has improved the economic and process efficiencies of biomass production on human urine (Yang et al., 2011; Chatterjee et al., 2019), there are still challenges in bringing this idea into fruition, including low-cost recovery of microalgae cells, high-efficient extraction of the biofuel, and scale-up of the photobioreactors.

To this end, a comprehensive understanding of the fundamentals, opportunities and perspectives of microalgae production in urine is of great significance. This Review sheds light on the advancements in microalgae cultivation on synthetic and human urine in photobioreactors. We systematically summarized the matrices that describe the algal growth and nutrient removal/recovery. Specific consideration has been given to the critical operating parameters influencing the process performance. Finally, we discuss the perspectives that emphasize the future research interest in the field.

## Urine, microalgae and photobioreactors

### Compositions of synthetic and natural urine

Human urine is composed of eight main ionic/non-ionic species (i.e., Na^+^, K^+^, Ca^2+^, Cl^−^, SO_4_^2−^, H_2_PO_4_^−^/HPO_4_^2−^, HCO_3_^−^ and urea; Golder et al., 2007). Most nitrogen in fresh urine originates from urea, which can be hydrolyzed into free ammonia (NH_3_), ammonium (NH_4_^+^) and bicarbonate (HCO_3_^−^) during storage. Both urea and ammonium are known to support the growth of microalgae (Tuantet, 2015). However, the free ammonia in urine can inhibit the growth of microalgae (Tuantet et al., 2013), and cell death may occur in the presence of high ammonia concentrations (Belkin and Boussiba, 1991). Piltz and Melkonian (2017) found satisfactory algal growth for all dilutions but not for undiluted urine. Thus, diluting synthetic or natural urine is essential to reduce ammonia’s toxic effect (Azov and Goldman, 1982). Cultivating microalgae in urine has two scenarios. The first scenario is a continuous culture by adding fresh urine as a daily nutrient stock (Tuantet et al., 2013). A process parameter, dilution rate (*D*, h^−1^), is introduced to express the relationship between the flow of the raw urine (*q*, L h^−1^) and the volume of the bioreactor (*V*, L). The other is a batch cultivation by employing diluted urine at a lower light intensity (Jaatinen et al., 2016), in which a parameter of dilution (or dilution ratio) is used (Tuantet et al., 2019).

#### Synthetic urine

Nitrogen sources in synthetic urine generally include ammonium, nitrate (hydrolysis) and urea. Hulatt et al. (2012) showed that the growth of *C. vulgaris* in a medium containing urea as the nitrogen source had a slightly higher maximum rate and yield than that with nitrate; however, no growth was observed with ammonium. The proposed explanation related to the ammonium utilization causing a significant decrease in the medium pH (e.g., from 6.8 to 4.0), thus resulting in the cessation of microalgae growth (Hulatt et al., 2012). In contrast, according to the earlier experimentation conducted by Schuler et al. (1953), *C. vulgaris* may prefer ammonium over nitrate as the nitrogen source. Trace elements added to synthetic urine include ethylene diamine tetraacetic acid (EDTA) ferric sodium salt, H_3_BO_3_, Mn(II), Zn(II) and Cu(II) (Tuantet, 2015). Chang et al. (2013) indicated that both fresh and synthetic urine might sustain rapid algal growth if additional trace elements such as Cu, Fe, Mn and Zn were dosed. Examples of synthetic urine used in microalgae cultivation are summarized in Table 1. As shown, the synthetic urine is commonly diluted 10–20 times upon use in batch studies, reducing ammonium inhibition while providing sufficient nutrients for microalgae growth. In comparison, *C. sorokiniana* could grow on pure urea and the algae growth would not be inhibited by ammonium up to a concentration of 1,400 mg NH_4_^+^-N L^−1^ at pH lower than 8.0 (Tuantet et al., 2013).

**Table 1:** Compositions of cultivation media used for microalgae.

Cultivation devices	Cultivation media	N source^1^ or hydrolyzed state^2,^^a^	TN or NH_4_^+^-N,^b^ mg L^−1^	TP or PO_4_^3−^-P, mg L^−1^	COD, mg L^−1^	Dilution	Refs.
Algal tanks	RHU	Unhydrolyzed^2^	11,450/500	850/n.a.	n.a.	50	Adamsson (2000)
24-well microtiter plates	SHU	Urea^1^	5,370/n.a.	n.a./733	1,428	5, 10	Tuantet et al. (2013)
	RHU	Unhydrolyzed^2^	6,340/442	401/n.a.	7,480	5, 10	
	RHU	Unhydrolyzed^2^	6,500/361	510/n.a.	6,305	5, 10	
Blue cap bottle	RHU	n.a.	4,850 ± 1,730/n.a.	155 ± 65/n.a.	n.a.	3/4, n.d., 2, 4	Zhang et al. (2014)
Erlenmeyer flasks	SHU	Urea^1^	305/n.a.	21.2/n.a.	n.a.	n.d.	de Wilt et al. (2016)
	RHU	Hydrolyzed^2^	5,124/3,240	151.2/n.a.	n.a.	6	
	SHU	Urea^1^	109.8/n.a.	13.0/n.a.	n.a.	n.d.	Yang et al. (2008)
	RHU	Unhydrolyzed^2^	4320/275	355/n.a.	n.a.	25, 75, 100, 150, 300	Jaatinen et al. (2016)
	RHU	Unhydrolyzed^2^	6,800/520	670/n.a.	n.a.	n.d.,100	
Transparent flasks	Livestock	Ammonium^1^	15.4 ± 0.3/13.4 ± 0.5	0.7 ± 0.0/n.a.	111.9 ± 16.7	n.d.	Kim and Kim (2017)
Outdoor raceway	RHU	Hydrolyzed^2^	3,480 ± 130/1,800 ± 750	190 ± 52/n.a.	5,500 ± 200	n.d., 2, 3, 4, 5, 10, 15, 20, 25	Chatterjee et al. (2019)
Tubular/bubble column PBR^c^	Piggery	Ammonium^1^	162.0 ± 8.0/n.a.	209.0 ± 5.5/n.a.	3,700 ± 51	n.d.	Zhu et al. (2013)
	Swine	Ammonium^1^	510 ± 10/460 ± 15	76.1 ± 5.0/36.7 ± 7.3	5,200 ± 900	2	Chen et al. (2020)
	SHU	Ammonium^1^	n.a./6,000	600/n.a.	n.a.	120	Chang et al. (2013)
	RHU	Hydrolyzed^2^	8,000 − 10,000/2,500–8,100	700–2,000/n.a.	8,000–10,000	120	
	RHU	Hydrolyzed^2^	8,880/6,000	792/n.a.	9,960	120	
	SHU	Urea^1^	109.8/n.a.	13.0/n.a.	n.a.	n.d.	Yang et al. (2008)
Flat/Panel PBR	Swine	Ammonium^1^	501.27	39.12	321.4	2	Cheng et al. (2020)
	SHU	Ammonium^1^	4,326/4,005	n.a./255	n.a.	2–50	Tuantet et al. (2014)
	RHU	Unhydrolyzed^2^	7,167/844	466/n.a.	8,349	2, 3, 5, 10, 20, 50	
	RHU	Unhydrolyzed^2^	4,358/393	200/n.a.	2,886		
	RHU	Hydrolyzed^2^	5,310/4660	260/n.a.	5,160		
	SHU	Ammonium^1^	6,990/n.a.	n.a./620	n.a.	1.8–10	Tuantet (2015)
	RHU	Unhydrolyzed^2^	2,260/312	n.a./215	2,520	5, 10, 20	
	RHU	Hydrolyzed^2^	3,550/733	n.a./387	4,885	5, 10, 20	
	RHU	Hydrolyzed^2^	3,500/3,260	n.a./341	3,555	5, 10, 20	
	RHU	Hydrolyzed^2^	2,220/2,150	n.a./295	2,795	5, 10, 20	
	RHU	n.a.	2,626/n.a.	146/n.a.	3,270	1.8–8.5	Tuantet et al. (2019)
MPBR	RHU	Hydrolyzed^2^	5,015 ± 209/2258 ± 43	345 ± 2/n.a.	n.a.	30	Nguyen et al. (2021)
Twin-layer PBR	RHU	n.a.	5,760/n.a.	290/n.a.	n.a.	n.d., 5, 10	Piltz and Melkonian (2017)
	RHU	n.a.	3,700/n.a.	210/n.a.	n.a.		
	RHU	n.a.	2,500/n.a.	170/n.a.	n.a.		

a Without specification, ammonia/ammonium insignificantly (~10%) contributes to the total nitrogen in unhydrolyzed human urine; SHU, syntenic human urine; RHU, real human urine; ammonium indicates that ammonium was used as N source, and urea indicates that urea was used as N source. n.a. represents “not available”. Superscript numbers, 1: N source in synthetic urine including urea and ammonium. 2: Whether the human urine was hydrolyzed before the tests.

b Concentrations shown in the table were the values before dilution; n.d, no dilution; TN, total nitrogen; TP, total phosphorus; COD, chemical oxygen demand.

c PBR, photobioreactor; MPBR, membrane photobioreactor.

#### Human urine

At 20°C, hydrolysis of ~64% of the urea in fresh human urine to ammonia and carbon dioxide occurs within 4 days (Adamsson, 2000). When hydrolyzed urine instead of fresh urine is used for cultivation, additional elements (e.g., Mg) may be required. In most cases, when urine is hydrolyzed, the N/P ratio increases due to the precipitation of phosphate (Chang et al., 2013; Tuantet et al., 2013). The compositions of real human urine samples are presented in Table 1. Similar to the problem existing in synthetic urine, microalgae growth in real urine suffers from inhibition from salt and nitrogen compounds, especially when free ammonia concentration is above 140 mg L^−1^ and/or nitrate concentration above 1,000 mg L^−1^ (Larsen et al., 2021). In batch studies, the dilution of human urine for microalgae cultivation is 5–100. While the proportion of viable cells in the biomass produced from less diluted urine was allegedly higher due to the abundant nutrients (Jaatinen et al., 2016), the chemistry condition in concentrated urine would prompt the precipitation of some critical elements (e.g., Fe^2+^, a component required for the synthesis of chlorophyll), thereby decreasing the overall microalgal growth and nutrient accumulation in the biomass (Tuantet et al., 2013). For example, in batch tests, *Scenedesmus* could grow at 10-time dilution (i.e., an initial NH_3_-N concentration of ~200 mg L^−1^) but not at lower dilutions, however, sustainable growth was only observed at 20-time dilution (at ~100 mg NH_3_-N L^−1^; Chatterjee et al., 2019). Thus, applying an optimized pre-dilution to pure urine is crucial before microalgae cultivation in a photobioreactor (Tuantet et al., 2019). In continuous cultivation, in contrast to the optimum dilution rate for synthetic urine ranging between 0.10 and 0.15 h^−1^, the algal growth in human urine demonstrates a higher efficiency at a dilution rate of 0.05 h^−1^ than at 0.10 h^−1^ (Tuantet, 2015). While biomass productivity could be higher at a dilution rate between 0.1 and 0.2 h^−1^, the nitrogen removal efficiency is compromised (Tuantet et al., 2019). In addition, an investigation has been conducted to assess whether differences in gender-related components, e.g., sex hormones, can influence the microalgae growth (Tuantet, 2015). Results showed that the impact was insignificant. Contamination of human urine by bacteria and the unmetabolized drug is another challenge for microalgae cultivation. Various bacteria were detected in *C. vulgaris* cultures employing sterilized or non-sterilized media, indicating that bacteria may play an essential role in microalgae growth in urine (Lakaniemi et al., 2012). As for trace drugs, pretreatment of urine with activated carbon can eliminate the potentially harmful effects (Piltz and Melkonian, 2017).

### Microalgae species

**Figure 2:**
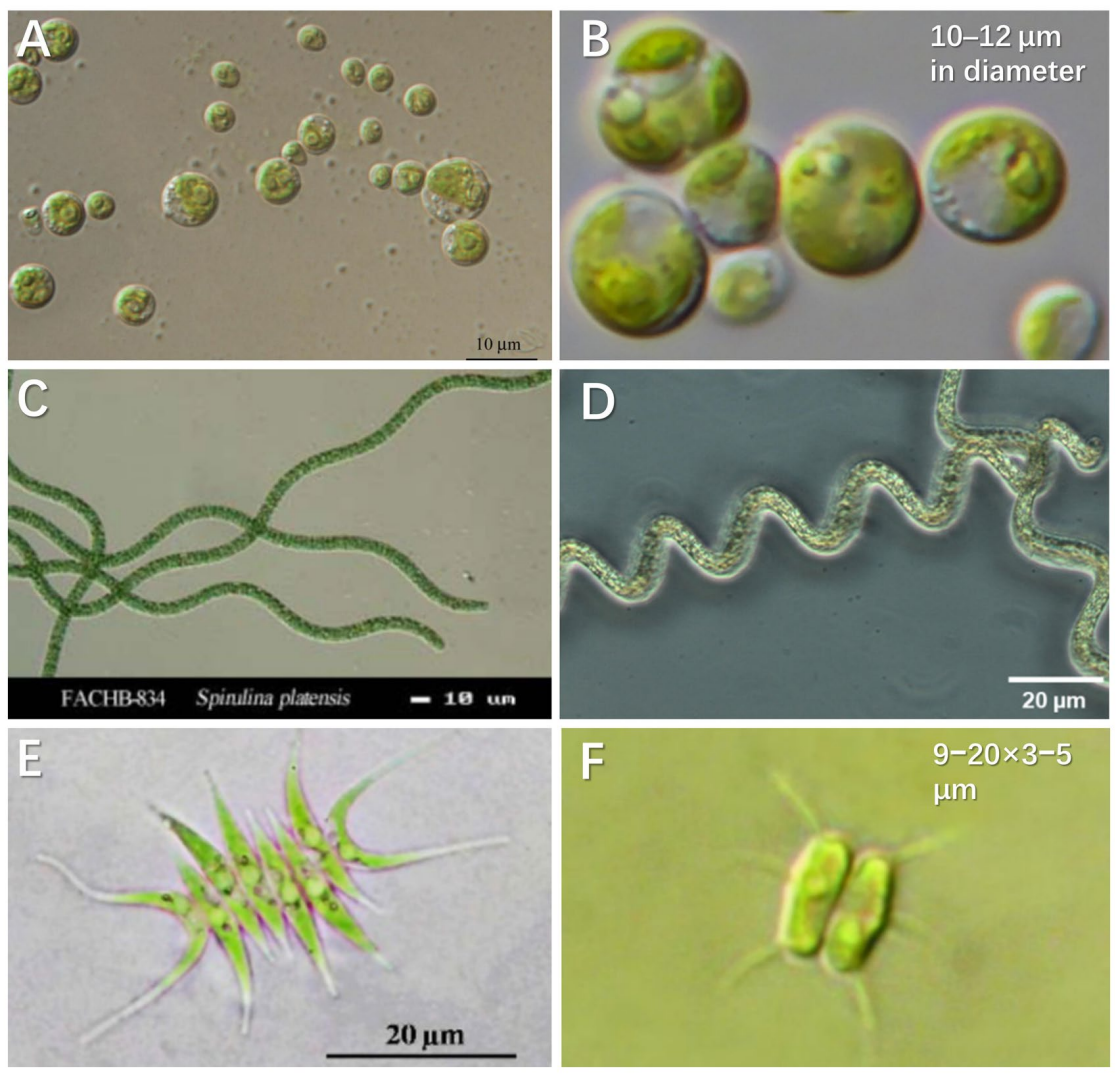
Microalgae species cultured in synthetic and real human urine. **(A)** *Chlorella sorokiniana* (CCAP 211/8K). Copyright Organisms (2020). **(B)** *Chlorella vulgaris* (CCAP 211-11b). Reproduced from Darienko et al. (2019) with permission. Copyright 2019 Taylor & Francis Group. **(C)** *Spirulina platensis* 834. Copyright Hydrobiology, F.A.C.C.O.T.I.O (2013). **(D)** *Arthrospira platensis*. Reproduced from Braune et al. (2021) with permission. Copyright 2021 MDPI. **(E)** *Scenedesmus acuminatus*. Adapted from Unpaprom et al. (2015) with permission. Copyright 2015 Science Publishing Group. **(F)** *Desmodesmus abundans.* Reproduced from Karlson et al. (2020) with permission. Copyright 2020 Swedish Biodiversity Data Infrastructure.

*Via* uptake and conversion of nutrients, microalgae enable urine purification and resource recovery in biofuels, biochemicals, and bio-fertilizer (Tuantet, 2015). As for biomass production, microalgae can grow autotrophically by utilizing organic/inorganic nitrogen and CO_2_/bicarbonate as carbon sources. Meanwhile, microalgae cells can directly use phosphate in urine under aerobic conditions and transform it into adenosine triphosphate (ATP) or other organic substances through assimilation and proliferation (Yang et al., 2008). Figure 2 presents the microalgae species successfully cultivated in synthetic and real human urine. Some typical species and their characteristics are summarized as follows:

*Chlorella* is a fast-growing microalgae species with reported maximum specific growth up to 0.25 h^−1^ under autotrophic and light saturating conditions (Cuaresma et al., 2009), the dry weight of which is contributed by 6%–8% nitrogen and 1%–2% phosphorus. Microalgae biomass of *Chlorella* can be directly used as a fertilizer and/or a potential source for chemicals and biofuels (Tuantet et al., 2013). Because *Chlorella* is rich in protein (Adamsson, 2000), the cultivated biomass from urine can be directly fed to zooplankton and the latter can be provided to fish in the constructed food chain (Adamsson, 2000). The widely investigated *Chlorella* in urine media includes *C. sorokiniana* and *C. vulgaris* (Figures 2A,B)*. C. vulgaris* has higher Omega-3 fatty acids content. These fatty acids are commonly found in green leafy vegetables and oily fish such as herring, sardines, and tuna (Revellame et al., 2021). *C. vulgaris* prefers ammonium to nitrate as a source of nitrogen, which benefits the growth in urine (Schuler et al., 1953). Another species of *Chlorella*, *C. sorokiniana* shows great potential for commercial production as a nutrient substitute for humans and animals (Morais Junior et al., 2020). *C. sorokiniana* is mesophilic, the growth of which is characterized by a relatively high optimal temperature (30°C–40°C) and a maximum specific growth rate of ~0.27 h^−1^ (Cuaresma et al., 2009). Furthermore, *C. sorokiniana* can synthesize neutral oil as triacylglycerol under stress conditions (e.g., under high light intensity or nutrient deficiency). The study by Tuantet et al. (2014) demonstrated that dilution of urine by two times could achieve adequate incubation of *C. sorokiniana* CCAP211/8K, which contributed to >90% of total nitrogen and phosphorus removal. Of all the commercial microalgae, while *Chlorella* has the second largest annual production inferior to *Spirulina*, the price of *Chlorella* is significantly higher on the market (Yuan et al., 2022).

*Spirulina* is the benchmark of microalgal biotechnology and is currently the most (3,000 dry tons annually) commercially produced microalgae (Detrell et al., 2020). *Spirulina* (*Arthrospira platensis*; Figures 2C,D), one of the essential cyanobacteria, produces high concentrations of pigments (chlorophyll a and phycocyanin), fatty acids (i.e., *γ*-linolenic acid) and proteins (Gutierrez-Salmean et al., 2015). *A. platensis* contains a more balanced ratio of saturated to unsaturated fatty acids including Omega-3 and Omega-6 (Revellame et al., 2021). Moreover, *S. platensis* has a relatively high cell growth rate and requires an easy process for biomass recovery as a result of the filamentous cell structure. Studies have demonstrated that the cultivation of *S. platensis* is viable for waste purification or aquatic food production (Chang et al., 2013). For example, *S. platensis* was harvested after 7 days of incubation and percentage removal of 97.0% and 96.5% was, respectively, achieved for NH_4_^+^-N and total phosphorus in the urine at a 120-dilution (Chang et al., 2013).

*Scenedesmus acuminatus* is also widely used in the treatment of anaerobic digestion effluents and secondary domestic wastewater (Figures 2E,F), because of its capacity to grow at a high biomass concentration of 8–11 g L^−1^ (Posadas et al., 2015; Tao et al., 2017b). *Scenedesmus* is one of the high protein content species, which contains 50%–56% protein, 10%–17% carbohydrate and 12%–14% lipid (Raeisossadati et al., 2020). For example, a strain of *Desmodesmus* sp. QL96 isolated from Tibet, China contains 17 amino acids (including 7 essential ones; Cheng et al., 2021). The properties of *Desmodesmus* highlight its commercial merits in biomass cultivation from urine. *Scenedesmus species* have been reported to take up high concentrations of nitrogen (273 mg L^−1^) and phosphorus (58.8 mg L^−1^; Kim et al., 2015). *Scenedesmus* was grown in 0.5% diluted urine supplemented with EDTA and iron, and its maximum biomass density was about 133 mg-dry weight L^−1^.

### Architecture of photobioreactors

**Figure 3:**
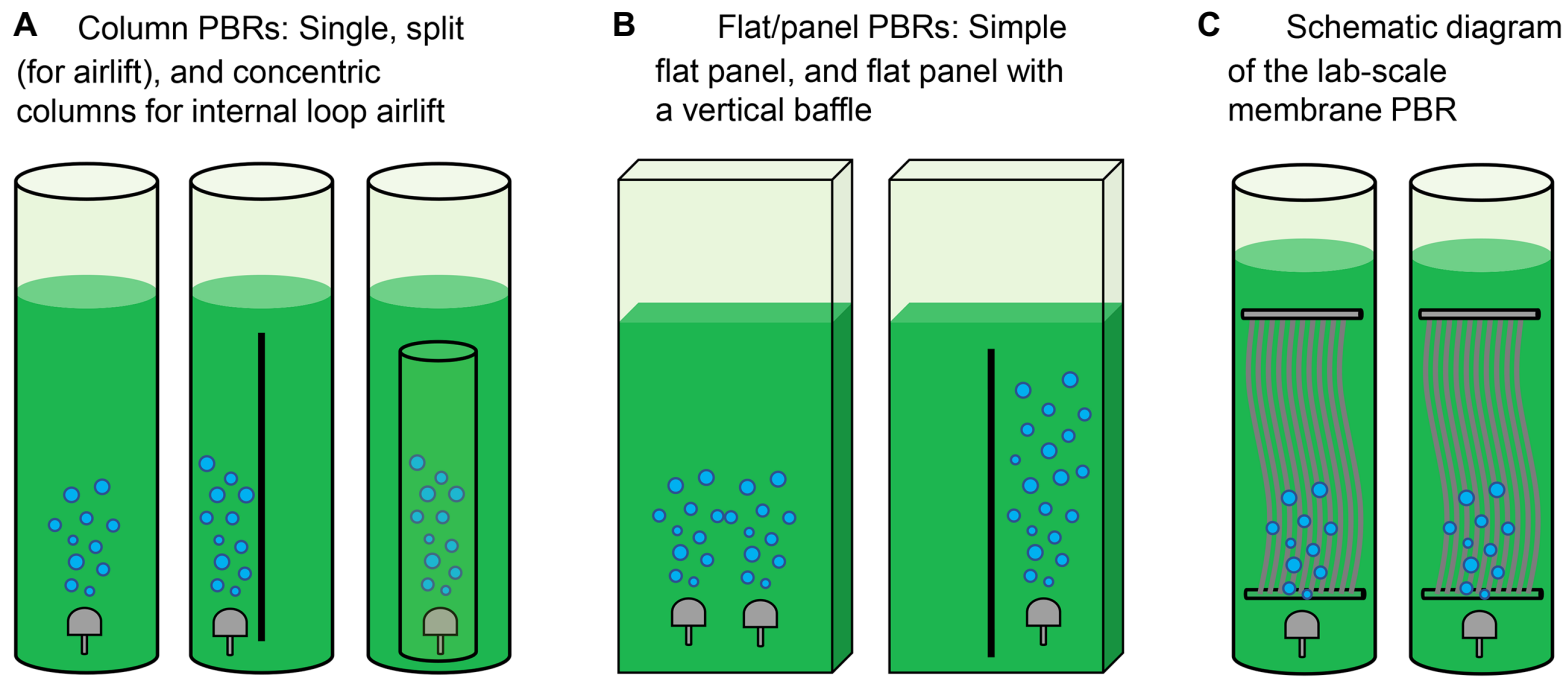
Architectures of photobioreactors (PBRs) for microalgae cultivation. **(A)** Column PBRs, **(B)** flat/panel PBRs, and **(C)** membrane PBR (MPBR). Refer to the study by Pawar (2016).

Microalgae are generally cultivated in open or closed systems (Song et al., 2018). Raceway ponds are traditional open systems to cultivate microalgae (Table 1). While these configurations have merits including low cost and simple operation, microalgal productivity in open systems is highly susceptible to environmental conditions. In comparison, closed systems including tubular, flat panel and bubble column photobioreactors (PBRs) are relatively costly and currently limited to small-scale microalgae cultures that generate high-value products including poly-unsaturated fatty acids, carotenoids and other chemicals for pharmaceutical and cosmetics industries (Rezvani et al., 2022). The operating cost for closed reactors relates to collecting culture fluid and microalgae cells. Recent advancements in reactor design have paved the way for more efficient enrichment and collection of microalgae (Zittelli et al., 2013; Tuantet, 2015). The architectures of PBRs for microalgae cultivation in urine are summarized in Figure 3 and Table 2.

**Table 2:** Summary of photobioreactors (PBR) and operating parameters for biomass culture in urine.

PBR types	Parameters of PBR	Illumination		Aeration	Light intensity	*T* (°C)	Refs
		Source	Switch				
Column PBR	n.a.^b^	Red (620 nm) LED	n.a.	1% CO_2_	300 μmol m^−2^ s^−1^	30	Yang et al. (2008)
	1.2 L (60 cm height, 7.2 cm diameter)	Concentric fluorescent lamp	14 h:10 h	CO_2_: 3 L min^−1^	444.4 W m^−2^^a^	30	Chang et al. (2013)
Flat/panel PBR	Flat panel with a vertical baffle (as shown by the right reactor): 0.925 L, 420 × 225 (mm)	High-pressure sodium lamps	n.a.	CO_2_: 5% v/v, 1, 0.76, 0.67 L L^−1^ min^−1^	490–1,550 × 2 μmol m^−2^ s^−1^	35/38	Tuantet et al. (2014)
	0.92 ± 0.12 L	High-pressure sodium lamps	n.a.	CO_2_: 8%–20%	n.a.	38	Tuantet (2015)
	0.90 L	High-pressure sodium lamps	12 h:12 h	CO_2_: 8%–20% v/v	1,530 μmol m^−2^ s^−1^	38	Tuantet et al. (2019)
Membrane PBR	Two PBRs with a diameter of 100 mm and a height of 600 mm, 4 L each. A hollow fiber membrane (MF) module (width × height = 95 mm × 320 mm, working surface area of 0.05 m^2^).	n.a.	24 h: 24 h	CO_2_ gas/air mixture at 2 L min^−1^ with 2.5% (v/v) of CO_2_.	3,000 lux	n.a.	Nguyen et al. (2021)

a 1 W m^−2^ = 4.5 μmol m^−2^ s^−1^.

b n.a. represents “not available.”

PBRs constructed for microalgae cultivation should sustain fast reaction rates, stable operation performance and a high capacity to recover nutrients *via* microalgae harvesting (Yang et al., 2008). In earlier experiments, Tuantet et al. (2013) cultured *Chlorella* on human and artificial urine microtiter plates. Column PBRs have simple configurations and better flow conditions (Figure 3A and Table 2). However, some practical problems exist when urine is used as feed. In addition to the nutrient levels of raw urine being unsuitable for algal growth, the dark color of urine and the low light penetration would challenge the PBR setup. To overcome the limitations, on the one hand, the bioreactors are typically fed with diluted urine. Conversely, an optimized system requires shorter light paths for microalgae growth. In the study by Chatterjee et al. (2019), extremely high dilution would be necessary even for only 50% nitrogen recovery in a 0.5 m deep raceway pond. To this end, a short light-path PBR (typically in the flat-panel configuration, Figure 3B) has been developed to supply light to all cells encapsulated inside the microalgal culture and thus support dense cultivation. Continuous microalgae cultivation has been carried out in a PBR with a narrow light path (i.e., 5 mm). However, an inherent conflict remained between the nitrogen removal and photosynthetic efficiency when the PBR was used to treat urine containing 0.77–2.6 g-N·L^−1^. Because an increase in the biomass concentration/density would shield the illumination and create a “dark zone” for a considerable part of the culture, advancements to minimize the “dark zone” in order to enhance biomass productivity are of significance in a short light-path PBR (Tuantet, 2015).

From a theoretical perspective, cultivation at a high cell density is essential to sustain high nutrient removal efficiencies in urine treatment (Tuantet et al., 2014). A PBR employing a higher hydraulic retention time (HRT) can prompt biomass growth and nutrient removal, but this may require a larger volume/footprint for reactor deployment. To address this issue, the membrane separation process has been integrated with PBR to increase the capacity and improve biomass recovery efficiency (Ma et al., 2017; Nguyen et al., 2021; i.e., membrane PBR in Figure 3C and Table 2). While membrane modules used in PBRs could be similar to those in conventional membrane bioreactors, the operating protocols may differ due to the characteristics of microalgae. For instance, the formation of a cake layer on the membrane surface in PBRs could be deemed as a means to recover the suspended cells from the dilute culture, which may pave the way for the application of dynamic membrane processes in membrane PBRs for more-efficient biomass harvesting (Ma et al., 2013b). Developing a realistic PBR with a compact structure is therefore one of the most critical interests in microalgae production in human urine (Larsen et al., 2021).

## Standard matrices to evaluate microalgae growth and nutrient removal/recovery

### Cell growth and biomass production

#### Specific growth rate

The specific growth rate (*μ*, h^−1^) of microalgae production in urine can be calculated by linear regression of the natural logarithm of optical density (OD) as a function of culturing time according to the following equation (Equation 1; Chang et al., 2013):


(1) $$\mu =\frac{\ln\left(\frac{N_{2}}{N_{1}}\right)}{t_{2}-t_{1}}$$

where *N*_1_ and *N*_2_ represent the OD of a predetermined wavelength [e.g., 750 nm (Tuantet et al., 2013)] at *t*_1_ and *t*_2_ respectively. For instance, the growth rates (0.20–0.38 day^−1^) of *C. vulgaris* cultured in urine at different dilutions (i.e., 1:25–1:300) of urine were determined using Equation 1, which were compared to that obtained in Chu-10 medium (0.37 day^−1^; Jaatinen et al., 2016). Note that either biomass (*X*, g L^−1^) or Chlorophyll *a* (Chl *a*) concentration (*C*_chl_, mg L^−1^) can be alternatively introduced into Equation 1 to estimate the specific growth rate (Porra et al., 1989).

#### Biomass productivity

The successful implementation of microalgae as a potential bio-energy feedstock depends on the biomass yield. For example, Yang et al. (2008) found that, in the batch culture of *Spirulina*, 1.05 g biomass could be obtained by treating 12.5 ml synthetic human urine. The standard parameter describing biomass productivity (*β*, g L^−1^ day^−1^) in batch reactors is given in Equation 2 (Gao et al., 2018; Nguyen et al., 2020a,b):


(2) $$\beta =\frac{X_{t}}{\mathrm{BRT}}$$

where *X*_t_ is the biomass concentration in a photobioreactor, and BRT is the biomass retention time (day; Chatterjee et al., 2019; Tuantet et al., 2019). *X*_t_ can be determined by employing the standard plate count method (counts ml^−1^), flow cytometry (counts ml^−1^), weighing of the volatile suspended solids (g L^−1^; Jaatinen et al., 2016) or calibration conversion of the OD values [i.e., *X*_t_ = 0.3421 × OD_750_ for *C*. *sorokiniana* (Zittelli et al., 2013; Tuantet, 2015)]. We summarized the *X*_t_ and *β* values describing algal production in different literature in Table 3, and the results indicated that a higher *β* (9.3–14.8 g L^−1^ day^−1^) was observed at low dilution (i.e., no dilution or dilution ratio = 2). Moreover, when a PBR is operated in continuous mode and at a steady state (i.e., when there is no accumulation of biomass in the reactor), the volumetric biomass productivity (*β*_vol_) is determined based on the biomass dry weight concentration (*X*_t_, g L^−1^) and the reactor dilution rate (*D*, h^−1^; Tuantet et al., 2019; Equation 3), which can be converted to the area productivity (*P*_area_) by integrating the reactor dimensions (Equation 4):


(3) $$\beta_{\mathrm{vol}}=X_{t}D$$

(4) $$P_{\mathrm{area}}=\frac{1000\beta_{\mathrm{vol}}}{\mathrm{SVR}}$$

where SVR is the surface area to volume ratio (m^−1^). When the microalgae community in urine is at the logarithmic phase, the net specific microalgae growth rate, d*X*/d*t*, is given in Equation 5:


(5) $$\frac{dX}{dt}=\left(\mu -D\right)X_{t}$$

**Table 3:** The performance of microalgae production in urine.

Microalgae	Cultivation media	Dilution	Biomass *X*_t_, g L^−1^	Productivity *β*, g L^−1^ day^−1^	Lipids, g L^−1^	Protein/Fatty acid content, %	Supplements	Refs.
*C. vulgaris*	RHU	n.d./100^a^	0.73/0.59VSS	0.08/0.06VSS	n.a.	n.a.	Trace elements	Jaatinen et al. (2016)
		25–300	0.48 − 0.60VSS	0.05–0.07VSS	n.a.	n.a.	–	
*C. sorokiniana*	RHU	6	1.8	n.a.	n.a.	n.a.	–	de Wilt et al. (2016)
			2.7	n.a.	n.a.	n.a.	With micropollutants	
	SHU	n.d.	5.5	n.a.	n.a.	n.a.		
*D. abundans*	RHU	n.d.	n.a.	14.5	n.a.	n.a.	–	Piltz and Melkonian (2017)
*C. soroliniana*	RHU	2	n.a.	14.8	0.9–3.6	38–48/(16 − 25w/w)	Mg and P	Tuantet et al. (2014)
	SHU	2	7.5	n.a.	n.a.	n.a.	P	
*S. acuminatus*	RHU	20 and 15	0.34	n.a.	n.a.	n.a.	–	Chatterjee et al. (2019)
*C. vulgaris*	RHU	30	2.14	0.313	n.a.	n.a.	–	Nguyen et al. (2021)
*S. platensis*	RHU	120	0.81	n.a.	19.8	35.4	–	Chang et al. (2013)
	SHU		0.75/1.17/1.75	n.a.	17.2/17.5/17.9	36.2/56.4/60.2	−/100/200 mg L^−1^ CH_3_COONa	
*S. platensis*	RHU/SHU	180	2.32/2.40	n.a.	20.43/17.58%	32.4/34.78	–	Feng and Wu (2006)
*C. soroliniana*	RHU	2	n.a.	9.3	16%–25% w/w	43%–53% w/w	Mg^2+^	Tuantet et al. (2013)
*S. platensis*	SHU/RHU	n.d.	1.05/2.9–3.4	n.a.	n.a.	n.a.	–	Yang et al. (2008)
*C. sorokiniana*	Swine^b^	2	5.54	n.a.	n.a.	0.27 g L^−1^ day^−1^	–	Chen et al. (2020)
		2	8.08	n.a.	n.a.	0.272 g L^−1^ day^−1^	–	

^a^n.a. represents “not available”; n.d., no dilution.

b Swine wastewater was listed for comparison.

#### Photosynthetic and harvesting efficiencies

Since the additional light source is widely implemented for microalgae production in urine, the observed biomass yield can be normalized to the lighting energy to assess the photosynthetic efficiency (*Y*_ph_, g mol_ph_^−1^; Equation 6):


(6) $$Y_{\mathrm{ph}}=\frac{X_{t}q}{I_{\mathrm{phin}24}A_{r}}=\frac{X_{t}q}{I_{\mathrm{phin}}\times 10^{-6}\times 24\times 3600\times A_{r}}$$

where *q* is the liquid flow (L day^−1^), *I*_phin24_ is the daily integral light intensity (mol_ph_ m^−2^ day^−1^), *A*_r_ is the reactor surface area (m^2^) and *I*_phin_ is the average light intensity (μmol_ph_ m^−2^·s^−1^). In the study by Tuantet et al. (2019), the maximum *Y*_ph_ of *C. sorokiniana* CCAP211/8 K reached 0.97 g mol_ph_^−1^ at a dilution rate of synthetic urine between 0.10 and 0.15 h^−1^, which is comparable to other studies using *C. sorokiniana* (Cuaresma et al., 2009; Holdmann et al., 2018). Following cultivation, settlement or separation processes (Tuantet et al., 2014) are applied to separate or recover microalgae cells from the dilute medium. The harvesting efficiency (*η*, %) is calculated by using Equation 7:


(7) $$\eta =\frac{X_{R}V_{R}}{X_{t}V}\times 100$$

where *X*_R_ is the biomass concentration in the recovered volume (*V*_R_). *V* is the liquid volume of the photobioreactor.

### Nutrients removal and recovery

#### Nutrient removal

Microalgae can absorb N and P nutrients into their cells at concentrations as low as 2.2 and 0.15 mg L^−1^, respectively (Boelee et al., 2011). When urine is used as a nutrient medium to cultivate microalgae, three routines of assimilation, ammonia volatilization, and denitrification, contribute to nitrogen removal (Gao et al., 2016). The percentage removal (*p*_N_, %) and removal rate (*r*_N_, mg L^−1^ day^−1^) of nitrogen are, respectively, determined by Equations 8, 9:


(8) $$p_{N}=\frac{C_{N,0}-C_{N,t}}{C_{N,0}}\times 100$$

(9) $$r_{N}=\frac{C_{N,0}-C_{N,t}}{t}\times 100$$

where *C*_N,0_ and *C*_N,t_ are the nitrogen concentrations (mg L^−1^) at *t* = 0 and time *t* (day). In comparison, phosphorus removal/recovery during microalgae cultivation on urine relates to both assimilation by microalgal cells and precipitation induced by pH changes (Singh et al., 2015; Wang et al., 2017). The calculations of percentage removal and removal rate of phosphorus also refer to Equations 8, 9.

**Figure 4:**
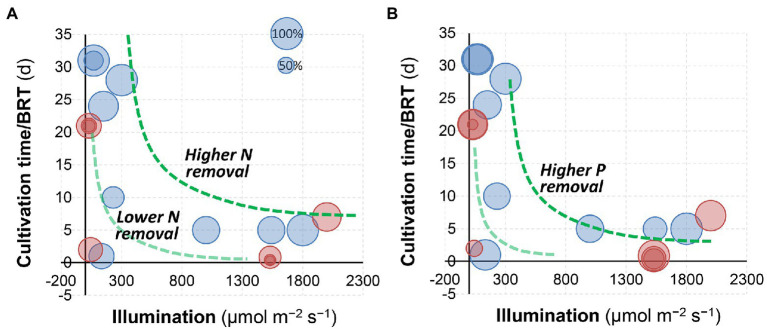
Comparison of **(A)** nitrogen and **(B)** phosphorus removal by algal cultivation at different illumination/light intensities and cultivation time. The size of bubbles represents the percentage removal. Red circles: dilution >20, and blue circles: dilution <10. Data were achieved from the literature (Adamsson, 2000; Yang et al., 2008; Chang et al., 2013; Tuantet et al., 2013, 2014, 2019; Zhu et al., 2013; Zhang et al., 2014; de Wilt et al., 2016; Jaatinen et al., 2016; Piltz and Melkonian, 2017; Chatterjee et al., 2019; Chen et al., 2020; Nguyen et al., 2021).

In a semi-continuous culture of *C. sorokiniana*, 84% removal of total nitrogen and nearly 100% removal of total phosphorus can be achieved *via* microalgae growth in fresh human urine (Zhang et al., 2014). Tuantet et al. (2014) demonstrated that the removal of nitrogen by microalgae cultivation (75%–85% or 1,000–1,300 mg L^−1^) was comparable with conventional treatment technologies including nitrification and anaerobic ammonia oxidation (Udert et al., 2003). Likewise, when *S. platensis* was applied to swine wastewater, it was reported that ammonia removal ranged from 84% to 96% (Chang et al., 2013). Figure 4 summarizes the nitrogen and phosphorus removal by microalgae cultivation from urine in different literature (Adamsson, 2000; Yang et al., 2008; Chang et al., 2013; Tuantet et al., 2013, 2014, 2019; Zhu et al., 2013; Zhang et al., 2014; de Wilt et al., 2016; Jaatinen et al., 2016; Piltz and Melkonian, 2017; Chatterjee et al., 2019; Chen et al., 2020; Nguyen et al., 2021). Generally, a higher illumination/light intensity and a longer cultivation time (or BRT) would result in higher N and P removal. In comparison, the impacts of dilution (red circles: dilution >20, blue circles: dilution <10) and photobioreactor configurations were less significant.

#### Nutrient recovery

Microalgae can use nitrate and nitrite to synthesize amino acids, proteins and other substances (Vílchez and Vega, 1995). Tuantet et al. (2014) showed that 85% of phosphorus and 90% of nitrogen could be recovered from urine by incorporation into biomass. Typically, the protein and lipid contents can be measured according to standard methods (Bahcegul et al., 2011; Chang et al., 2013) to estimate the conversion of nutrients in urine to biomass. For example, following cultivation in synthetic urine, the protein and lipid contents (% dry weight) obtained from *S. platensis* were 36.2% and 17.2%, respectively, (Chang et al., 2013), which were comparable with those (35.4% and 19.8%) obtained in real human urine. This was consistent with the conclusion drawn by Danesi et al. that the lipid content of *Spirulina* was not affected by the nitrogen source (Danesi et al., 2002). Data in Table 3 shows that the protein content (%) of the microalgae cultivated in urine generally ranges from ~35% to ~60% of the biomass with the lipid content varying in response to the medium composition and dilution.

## Critical operating factors that influence the cultivation efficiency

According to the literature review, operating parameters including the light intensity, temperature, retention time, dose of trace elements and carbon source, and solution pH significantly impact the microalgae growth and conversion of nutrients to biomass. It is vital to investigate and determine the optimal operating conditions to prompt microalgae growth and resource recovery (Kinnunen and Rintala, 2016).

### Light intensity and temperature

Light intensities and switch modes used for microalgae production in urine are summarized in Table 2. Essentially, light intensity influences the microalgae photosynthesis and consequently their growth rate *via* modulation of ATP and NADPH production and essential molecules synthesis. The study by Tuantet et al. (2013) indicated that illumination was one of the most important factors influencing algal growth and nutrients removal, which is also confirmed by the analysis in Figure 4. A biomass density of 6.6 g L^−1^ was obtained in 2-times diluted urine at a light intensity of 1,540 μmol m^−2^ s^−1^ compared to that of 3.8 g L^−1^ in 10-times diluted urine at 1050 μmol m^−2^ s^−1^ (Tuantet et al., 2014). Note that different illumination units (e.g., μmol m^−2^ s^−1^, W m^−1^ and lux) have been used in the literature (Yang et al., 2008; Chang et al., 2013; Tuantet et al., 2014, 2019; Tuantet, 2015), and the photosynthetic photon flux density (μmol m^−2^ s^−1^) could be converted to lux by multiplying a factor of 54–82 (for sunlight and high-pressure sodium lamps) according to the manufacturer (for example, Apogee Instruments, Inc., UT, United States). Overall, it was suggested that increasing the light intensity up to 1,500 μmol m^−2^ s^−1^ could prompt microalgae growth and improve the nutrient removal efficiency (Tuantet et al., 2014).

Temperature change also influences the solubilization and volatilization of ammonia, thus leading to pH excursion. As shown in Table 2, a temperature between 30°C and 40°C is typically applied to the incubation because, for example, the optimal temperature for the growth of *S. platensis* is between 29°C and 32°C (Yang et al., 2008). Nevertheless, some microalgae can tolerate low temperatures; for example, *S. acuminatus* could grow in human urine even at 5°C with the recovery of N and P achieving 52% and 38%, respectively, (Chatterjee et al., 2019).

### Retention time

As for photobioreactor design and operation, biomass and hydraulic retention time (BRT and HRT) are two key parameters (Nguyen et al., 2021). Results in Figure 4 indicate that BRT may have a positive relationship with nutrients removal from urine. In a PBR, BRT relates to biomass accumulation and thus determines the nutrient removal rates (Akerstrom et al., 2014). A short BRT (2–5 days) may not be sufficient to sustain the rapid growth of microalgae, which consequently limits the biomass production rate even when a high nitrogen uptake rate is obtained (Luo et al., 2017; Praveen et al., 2019). Likewise, the highest TP removal of 52.1% was achieved at a BRT of 7 days during incubation (Nguyen et al., 2021). It should be noted that a longer BRT may result in the deterioration of the settling/harvesting properties of the biomass (Wang et al., 2013; Ma et al., 2018a). In addition, a longer HRT resulting from an extended BRT would decrease the productivity of a PBR to treat human urine. Conversely, a short HRT leads to higher nutrient loads but compromises the removal of N and P. To address the limitations, membrane separation processes have been integrated with PBRs (MPBR) to achieve flexible control of BRT and HRT. High nutrient loads such as 90–110 mg-N L^−1^ day^−1^ and 5–6 mg-P L^−1^ day^−1^ have been used in MPBRs (Nguyen et al., 2021). In summary, the BRT and HRT should be set given the treatment efficiency and capital cost for bioreactor deployment. According to the study by Nguyen et al. (2021), a short BRT (7 days) and an extended HRT (>2 days) are thus suggested to capture nutrients from urine while minimizing the environmental impacts effectively.

### Trace elements

Tuantet (2015) found that the algal growth was inhibited due to the exhaustion of trace elements within 24 h, highlighting the importance of trace elements in biomass production in diluted urine. As for the effect of dilution on microalgae growth, Jaatinen et al. (2016) reported that the highest biomass densities of 0.73 and 0.60 g L^−1^ were obtained at 1:100 dilution of urine with and without the addition of trace elements. Moreover, *C. sorokiniana* showed the fastest growth rate in urine diluted 20 times with trace elements added (Tuantet et al., 2013).

Magnesium (Mg^2+^), iron (mainly in the form of Fe^II^) and certain trace elements are present in urine at low concentrations (Tuantet et al., 2013). When urine is collected and stored, the formation of precipitates due to an increase in pH reduces the availability of these elements. Udert et al. (2003) demonstrated that the precipitation capacity of guano stone and octa calcium phosphate in urine reached 87% when the hydrolysis rate was 11%. As a result, the magnesium content in hydrolyzed urine (0.15–0.17 mg L^−1^) could be significantly lower than in fresh urine (25.4 ± 17.0 mg L^−1^; Zhang et al., 2014). Mg^2+^ plays a vital role in algal metabolism because it is essential for chlorophyll production (Sydney et al., 2010). The magnesium content in *Chlorella* sp. ranged from 0.36% to 0.80% of dry weight, and only 40 mg L^−1^ dry biomass could be sustained at a 0.36% magnesium content (Borowitzka and Borowitzka, 1988). Therefore, magnesium supplementation is essential to promote microalgal growth in hydrolyzed urine (Table 3; Tuantet et al., 2019). No significant difference was found between the specific growth rates of microalgae fed with hydrolyzed urine with additional Mg^2+^ (*μ* = 0.095–0.111 h^−1^) as compared to synthetic and fresh urine (Tuantet et al., 2013). Moreover, iron is also one of the most crucial trace metals involved in the enzymatic reactions of photosynthesis in photosystem I (PSI) and PSII (Cao et al., 2014). An increase in the iron concentration in the medium (1.2 × 10^−2^ mM) would elevate the biomass as well as lipid content of *C. vulgaris* (Liu Z.-Y. et al., 2008). Note that significant elements such as Cl also indispensably contribute to the photosynthesis of chlorophyll and affect the uptake of trace elements (Yang et al., 2008).

### Inorganic/organic carbon source

Typically, microalgae production in human urine uses the internal inorganic carbon source of bicarbonate (HCO_3_^−^) and/or exogenous sparged CO_2_. Additional CO_2_ can prompt the growth of microalgae. As shown in Table 2, the gas concentration (v/v) of extra CO_2_ ranges from 1% to 20% because an overhigh CO_2_ dose may cause acidification of the media. Nevertheless, field studies have shown that microalgae cultivation by using flue gasses can withstand a high CO_2_ concentration of 40% (Pires et al., 2012).

Besides, microalgae can grow under mixotrophic conditions. Under autotrophic conditions, *S.platensis* fed with an inorganic carbon source (with ammonium or urea as the nitrogen source) demonstrated a yellowish-green appearance with relatively low protein content. At the same time, the biomass became green and difficult to settle down after adding an organic carbon source (Chang et al., 2013). Adding an organic carbon source could alleviate the inhibition effect of ammonium. For example, by adding 100 and 200 mg L^−1^ sodium acetate (or glucose) to the synthetic urine (Table 3), the productivity of *S.platensis* was improved with the nitrogen and phosphorus removal increasing from 97% and 96.5% to ~100% and 98%, respectively (Chang et al., 2013). As for treating urea in human urine, adding an organic carbon source could also facilitate the removal of ammonia *via* biomass production. As such, introducing waste organic carbon sources (e.g., effluents from food plants) to microalgae cultivation in human urine can provide a valuable solution to increase nutrient recovery efficiency.

### pH

**Figure 5:**
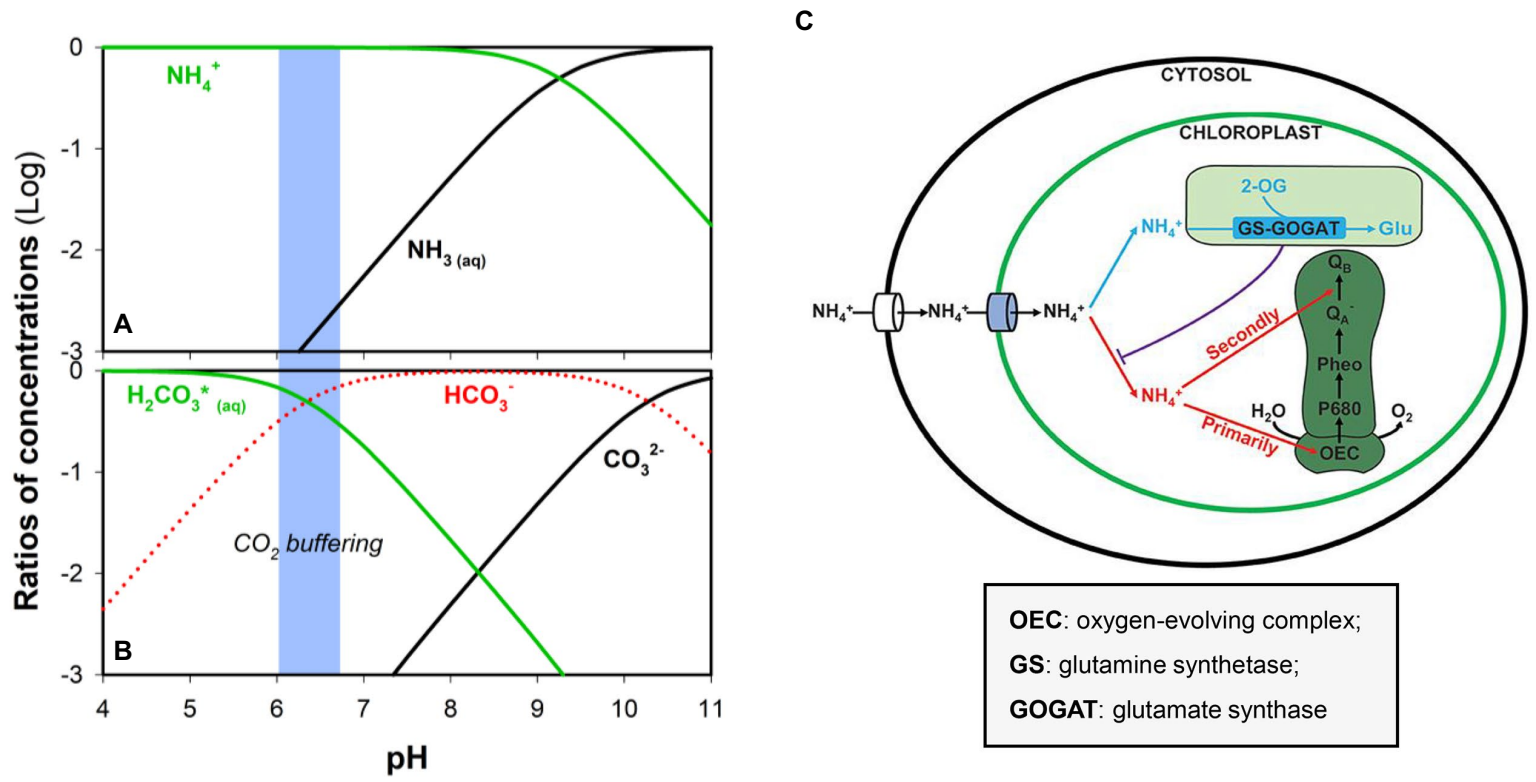
Ratios of **(A)** NH_4_^+^/NH_3_ and **(B)** H_2_CO_3(aq)_/HCO_3_^−^/CO_3_^2−^ concentrations at different pH. **(C)** Competition between assimilation and PSII damage by ammonium nitrogen in the chloroplast. Blue: N source by GS-GOGAT, and Red: hazardous material to photosynthesis, damaging the OEC and then blocking electron transport from Q_A_^−^ to Q_B_^−^. Reproduced from (Wang et al., 2019) with permission. Copyright 2022 Frontiers.

Medium pH directly influences microalgae growth and determines the speciation of nutrients that may support or inhibit biomass production. As aforementioned, hydrolysis of urea in human urine produces HCO_3_^−^ and ammonia while raising the solution pH (Adamsson, 2000; Zhang et al., 2013). Conversion between ammonia and ammonium is primarily determined by the solution pH (p*K*_a_ = 9.25; Zhang et al., 2018; He et al., 2022; Zhang et al., 2022), and higher pH could facilitate the transformation of NH_4_^+^ to NH_3_ (Figure 5). NH_3_ is far more toxic than NH_4_^+^ because the transport of NH_4_^+^ involves the participation of transporters (Figure 5C; Källqvist and Svenson, 2003; Wang et al., 2019). Ammonia or free ammonia can reduce photosynthetic activity and directly shows toxicity to microalgae (Zhang et al., 2014). The photosynthetic rate of *Scenedesmus* was allegedly decreased by 50% of its maximum rate in the presence of free ammonia of 20 mg L^−1^ under basic conditions (Azov and Goldman, 1982), though different microalgae species have different tolerance to the pH-dependent toxicity of NH_3_/NH_4_^+^ (Tao et al., 2017a). As mentioned above, a high pH level can lead to the precipitation of unchelated trace metals, thus inhibiting algal growth (Adamsson, 2000). To stabilize the solution pH and neutralize the alkalinity especially when human urine is directly used as the feed, sparging of CO_2_ (or diluted CO_2_) has been carried out. In addition to serving as an inorganic carbon source, the excessive CO_2_ can buffer the pH (p*K*_a_ (H_2_CO_3_/HCO_3_^−^) = 6.30; Ma et al., 2018b; Figure 5B) and prevent the inhibition of free ammonia on algal growth (Tuantet et al., 2014).

With the absorption of ammonium in algal growth, the pH would decrease because of the production and accumulation of H^+^, which slows down the growth rate (Azov and Goldman, 1982). In a photobioreactor, when the medium pH drops from pH 6.8 to <4 during cultivation, the microalgae would be subject to the cessation of growth or even death (Hulatt et al., 2012; Jaatinen et al., 2016). *C. sorokininana* has shown a high specific growth rate within the pH range from 4 to 7 (Tuantet et al., 2014). In addition, consideration should be given to the source of human urine. For example, it has been reported that gender may influence the pH because the female urine demonstrated a more narrow pH window (7.1–7.9) as compared to that (5.7 and 8.0) of the male urine (Tuantet, 2015).

## Perspectives and outlook

In this section, we discuss the challenges that should be addressed toward a broader application of microalgae production in urine. Important questions may include (i) the impacts of co-existing bacteria, and (ii) contamination by human metabolites. According to the literature, consideration should also be given to the genetic engineering that has been playing a more crucial role in increasing biomass/biofuel production.

### Co-existing bacteria

When non-sterilized human urine is used as the feed, bacterial contamination of the cultivations very likely occurs, which leads to competition for the nutrients and lower conversion to algal biomass. While this problem in the early growth stage may be solved by sterilization of the inoculation medium, it was reported that a large variety of bacteria were detected from *C. vulgaris* cultures grown on both sterilized and non-sterilized media at an extended cultivation period (Jaatinen et al., 2016). Maintaining the culture composition in the photoautotrophic mode (i.e., in the absence of organic carbon) may inhibit the competition from bacterial growth. Microalgal cells can regain dominance in the diverse community in 2 days when the organic carbon supply ceases (Zhang et al., 2014). Nevertheless, the autotrophic nitrifiers can adapt to fill a similar ecological niche compared to the microalgae though the biomass yield of ammonia-oxidizing bacteria is low (Ma et al., 2013a, 2015). In the symbiosis, a consortium of microalgae and nitrifying bacteria can decrease the need of expensive external aeration (Sun et al., 2020; Li et al., 2022). The photosynthetic oxygenation rate can drive the nitrification in urine with a volumetric nitrification rate of 67 mg N L^−1^ day^−1^, and a maximum biomass-specific photooxygenation rate of 160 mg O_2_ gVSS^−1^ day^−1^ (Muys et al., 2018).

In addition, the microalgae growth also leads to the secretion of extracellular organic matter that can be used by heterotrophic bacteria (Zhang et al., 2014). In turn, microalgae can convert nutrients into cellular components through photosynthesis and respiration. Currently, some single-cell microalgae such as *Chlamydomonas*, *Chlorella* and *Phormidium* are proven candidate for the formation of symbiosis suitable for waste treatment and facilitates the removal of N and P (Nguyen et al., 2020a). Inoculation of *Arthrospira platensis* with nitrated urine has been found to exhibit better growth and produce 62% more protein than untreated urine (Coppens et al., 2016). As for biofuel production, the biomass consisting of bacteria and microalgae may provide higher biogas production than pure microalgae (Jaatinen et al., 2016). For instance, 17%–24% higher methane yields (376–403 ml-CH_4_·g-*VS*^−1^) were obtained from a mixture of microalgae (*C. vulgaris*) and bacteria (1%–10%) than the control that only contained microalgae (Lu et al., 2013). As such, the following studies to explore (i) the algal and bacterial inter-group competition and collaboration and (ii) the impacts of co-existing bacteria on biofuel conversion are required to advance the process performance.

### Contamination by human metabolites

Source-separated urine contains about 60 ± 30% of drugs and lifestyle biomarkers consumed by humans (Monetti et al., 2022). Following intake, ~50% of pharmaceuticals do not change the chemical form, and are discharged with the intermediates as human metabolites (Lienert et al., 2007). Some human metabolites (e.g., conjugates of the antibiotic sulfamethoxazole, the anti-convulsant carbamazepine and the analgesic ibuprofen; Quinn et al., 2009; Ren et al., 2022a,b; Yang et al., 2022) have detrimental effects on the environment and may pose direct toxicity to microalgae. For instance, the anticonvulsant carbamazepine and the antidepressant fluoxetine form more toxic metabolites than their parent compounds (Verstraete et al., 1997; Jelic et al., 2015; Monetti et al., 2022). In contrast to the abundant studies of the contents of N, P and organic matter (Table 1) that influence microalgae growth and biomass production, there is little investigation of the micropollutants in urine involving the metabolism of microalgae. de Wilt et al. (2016) evaluated the efficiency of microalgae *C. sorokiniana* to remove six spiked pharmaceuticals (147 ± 9 μg L^−1^ diclofenac, 317 ± 33 μg L^−1^ ibuprofen, 337 ± 23 μg L^−1^ paracetamol, 181 ± 62 μg L^−1^ metoprolol, 117 ± 17 μg L^−1^ carbamazepine and 202 ± 30 μg L^−1^ trimethoprim). Results showed that 60%–100% of diclofenac, ibuprofen, paracetamol and metoprolol could be readily removed by photolysis and biodegradation while carbamazepine was refractory (removal <30%; de Wilt et al., 2016). While the presence of micropollutants at 100–300 μg L^−1^ did not inhibit microalgae (*C. sorokiniana*) growth, the deployment of pre-treatment technologies may be required at higher concentrations to prevent the pitfalls. Specific micropollutants can be removed by activated carbon, an effective absorbent for various organic and inorganic molecules because of the large surface area, porous structure and surface-bound groups (Yin et al., 2007). Activated carbon adsorption has been applied to eliminate antibiotics, beta-blockers, and nonsteroidal anti-inflammatory drugs from urine (Udert et al., 2016). Future work is essential to (i) assess the microalgae response to micropollutants at elevated concentrations, and (ii) develop cost-effective, reliable and environmentally benign processes to polish human urine under realistic conditions.

### Genetic engineering

Essentially, the accumulation of neutral lipids by *Acutodesmus*, *Phaeodactylum*, *Dunaliella* and *Nannochloropsis* requires nutrient limitation or starvation, which can inhibit microalgal growth (Sun et al., 2018). While the acquisition of favorable and stable traits can be conducted *via* crossbreeding for crops (Armbrust, 1999; Chepurnov et al., 2008), this is not applicable to most microalgae that have some deficiency (Dismukes et al., 2008). To address the limitation, microalgae genetic engineering is considered as an optimal approach to solve this bottleneck. Direct or indirect genetic modification has been proposed as a means to improve the growth and lipid productivity of promising microalgal strains.

Genetic engineering can facilitate lipid accumulation without affecting the algal growth (Munoz et al., 2021), by modifying the single metabolic pathways including fatty acid synthesis metabolism, Kennedy pathway, polyunsaturated fatty acid and triacylglycerol metabolisms and fatty acid catabolism. For example, the synthesis of fatty acids requires a continuous supply of acetyl-CoA. Compared with wild type strains, the total lipid contents of *N. oceanica* and *Schizochytrium* sp. were increased by 36% and 11%, respectively, by employing the overexpress of the malonyl-CoA acyl carrier protein transacylase (Chen et al., 2017). In addition, commercial production of large-scale bulk of microalgae is still not feasible (Remmers et al., 2018). Genetic engineering may pave the way for increasing the photosynthetic rate to modulate the carbon flux toward lipids while maintaining high biomass production (Ajjawi et al., 2017). This strategy to prompt the yield of microalgae lipids may eventually make microalgal derivatives an effective means of commercial biofuels.

## Conclusion

In conclusion, we present a comprehensive overview of the synthetic/human urine, microalgae species and photobioreactors that have been recently used in the algal production in urine. The standard matrices, including specific growth rate, biomass productivity, photosynthetic and harvesting efficiencies, and nutrients removal and recovery, have provided a platform for comparison among different studies. A summary of the critical operating factors is expected to facilitate our understanding of how the photobioreactors should be maintained to achieve high efficiencies. It is also recognized that the impacts of co-existing bacteria, contamination by human metabolites, and genetic engineering require continuing investigation toward a broader application of microalgae biomass production in urine.

## Author contributions

YT: resourcing and writing—original draft preparation. ZL: conceptualization and resourcing. JuZ: reviewing and editing. JiZ: data collection. DH: writing—reviewing and editing. JM: supervision, writing—reviewing, and editing. All authors contributed to the article and approved the submitted version.

## Funding

This work was financially supported by the National Natural Science Foundation of China (52100030), the Guangdong Natural Science Funds for Distinguished Young Scholars (2022B1515020053) and the Guangzhou Science and Technology Project (202201010622).

## Conflict of interest

The authors declare that the research was conducted in the absence of any commercial or financial relationships that could be construed as a potential conflict of interest.

## Publisher’s note

All claims expressed in this article are solely those of the authors and do not necessarily represent those of their affiliated organizations, or those of the publisher, the editors and the reviewers. Any product that may be evaluated in this article, or claim that may be made by its manufacturer, is not guaranteed or endorsed by the publisher.

## Figures and Tables

**Table:** 

ATP	Adenosine triphosphate	
BRT	Biomass retention time	
GOGAT	Glutamate synthase	
GS	Glutamine synthase	
HRT	Hydraulic retention time	
MF	Microfiltration	
MPBR	Membrane photobioreactor	
OD	Optical density	
OEC	Oxygen-evolving complex	
PBR	Photobioreactor	
PSI	Photosystem I	
PSII	Photosystem II	
PSBR	Porous substrate photobioreactors	
SVR	Surface area to volume ratio	
TN	Total nitrogen	
TP	Total phosphorus	
**Symbol**	**Entity**	**Unit**
*P*_area_	Area productivity	g m^−2^ day^−1^
*I*_phin_	Average light intensity	μmol_ph_ m^−2^·s^−1^
*X*	Biomass concentration	g L^−1^
*X*_R_	Biomass concentration in the recovered volume	g L^−1^
BRT	Biomass retention time	day
*β*	Biomass productivity	g L^−1^ day^−1^
Chl *a*	Chlorophyll *a* concentration	mg L^−1^
*I*_phin24_	Daily integral light intensity	mol_ph_·m^−2^·day^−1^
*D*	Dilution rate	h^−1^
*η*	Harvesting efficiency	%
HRT	Hydraulic retention time	day
*q*	Liquid flow	L day^−1^
*C*_N_	Nitrogen concentrations	mg L^−1^
*N*	OD of a predetermined wavelength	abs
*p*_N_	Percentage removal of nitrogen	%
*Y*_ph_	Photosynthetic efficiency	g mol_ph_^−1^
*A*_r_	Reactor surface area	m^2^
*r*_N_	Removal rate of nitrogen	mg L^−1^ day^−1^
*μ*	Specific growth rate	h^−1^
SVR	Surface to volume ratio	m^−1^
*t*	Time	day (or h)
*V*_R_	Volume of the recovered solution	L

## References

[ref1] Adamsson M. (2000). Potential use of human urine by greenhouse culturing of microalgae (*Scenedesmus acuminatus*), zooplankton (Daphnia magna) and tomatoes (Lycopersicon). Ecol. Eng. 16, 243–254. doi: 10.1016/S0925-8574(00)00064-1

[ref2] Ajjawi I. Verruto J. Aqui M. Soriaga L. B. Coppersmith J. Kwok K.. (2017). Lipid production in *Nannochloropsis gaditana* is doubled by decreasing expression of a single transcriptional regulator. Nat. Biotechnol. 35, 647–652. doi: 10.1038/nbt.3865, PMID: 28628130

[ref3] Akerstrom A. M. Mortensen L. M. Rusten B. Gislerod H. R. (2014). Biomass production and nutrient removal by *Chlorella* sp. as affected by sludge liquor concentration. J. Environ. Manag. 144, 118–124. doi: 10.1016/j.jenvman.2014.05.015, PMID: 24935023

[ref4] Armbrust E. V. (1999). Identification of a new gene family expressed during the onset of sexual reproduction in the centric diatom *Thalassiosira weissflogii*. Appl. Environ. Microbiol. 65, 3121–3128. doi: 10.1128/AEM.65.7.3121-3128.1999, PMID: 10388712 PMC91465

[ref5] Azov Y. Goldman J. C. (1982). Free ammonia inhibition of algal photosynthesis in intensive cultures. Appl. Environ. Microbiol. 43, 735–739. doi: 10.1128/aem.43.4.735-739.1982, PMID: 16345983 PMC241910

[ref6] Bahcegul E. Tatli E. Haykir N. I. Apaydin S. Bakir U. (2011). Selecting the right blood glucose monitor for the determination of glucose during the enzymatic hydrolysis of corncob pretreated with different methods. Bioresour. Technol. 102, 9646–9652. doi: 10.1016/j.biortech.2011.07.116, PMID: 21880485

[ref7] Behera B. Patra S. Balasubramanian P. (2020). Biological nutrient recovery from human urine by enriching mixed microalgal consortium for biodiesel production. J. Environ. Manag. 260:110111. doi: 10.1016/j.jenvman.2020.110111, PMID: 32090822

[ref8] Belkin S. Boussiba S. (1991). High internal pH conveys ammonia resistance in *Spirulina platensis*. Bioresour. Technol. 38, 167–169. doi: 10.1016/0960-8524(91)90149-E

[ref9] Boelee N. C. Temmink H. Janssen M. Buisman C. J. Wijffels R. H. (2011). Nitrogen and phosphorus removal from municipal wastewater effluent using microalgal biofilms. Water Res. 45, 5925–5933. doi: 10.1016/j.watres.2011.08.044, PMID: 21940029

[ref001] Borowitzka M. A. Borowitzka L. J. (1988). Micro-algal biotechnology. Cambridge University Press.

[ref10] Braune S. Krüger-Genge A. Kammerer S. Jung F. Küpper J. H. (2021). Phycocyanin from arthrospira platensis as potential anti-cancer drug: review of in vitro and in vivo studies. Life 11:91. doi: 10.3390/life11020091, PMID: 33513794 PMC7911896

[ref11] Cao J. Yuan H. Li B. Yang J. (2014). Significance evaluation of the effects of environmental factors on the lipid accumulation of Chlorella minutissima UTEX 2341 under low-nutrition heterotrophic condition. Bioresour. Technol. 152, 177–184. doi: 10.1016/j.biortech.2013.10.084, PMID: 24291318

[ref12] Chang Y. Y. Wu Z. C. Bian L. Feng D. L. Leung D. Y. C. (2013). Cultivation of Spirulina platensis for biomass production and nutrient removal from synthetic human urine. Appl. Energy 102, 427–431. doi: 10.1016/j.apenergy.2012.07.024

[ref13] Chatterjee P. Granatier M. Ramasamy P. Kokko M. Lakaniemi A. M. Rintala J. (2019). Microalgae grow on source separated human urine in Nordic climate: outdoor pilot-scale cultivation. J. Environ. Manag. 237, 119–127. doi: 10.1016/j.jenvman.2019.02.074, PMID: 30784859

[ref14] Chen C. Y. Kuo E. W. Nagarajan D. Ho S. H. Dong C. D. Lee D. J.. (2020). Cultivating Chlorella sorokiniana AK-1 with swine wastewater for simultaneous wastewater treatment and algal biomass production. Bioresour. Technol. 302:122814. doi: 10.1016/j.biortech.2020.122814, PMID: 32004812

[ref15] Chen J. W. Liu W. J. Hu D. X. Wang X. Balamurugan S. Alimujiang A.. (2017). Identification of a malonyl CoA-acyl carrier protein transacylase and its regulatory role in fatty acid biosynthesis in oleaginous microalga *Nannochloropsis oceanica*. Biotechnol. Appl. Biochem. 64, 620–626. doi: 10.1002/bab.1531, PMID: 27572053

[ref16] Cheng H. Li G. Han Z. Li J. Wamg F. (2021). Evaluation of Desmodesmus sp. QL96 protein on nutritional value and its physiological Activitiy. Chin. J. Tropical Crops 42:1440. doi: 10.3969/j.issn.1000-2561.2021.05.033

[ref17] Cheng P. Osei-Wusu D. Zhou C. Wang Y. Xu Z. Chang T.. (2020). The effects of refractory pollutants in swine wastewater on the growth of *Scenedesmus* sp. with biofilm attached culture. Int. J. Phytoremediation 22, 241–250. doi: 10.1080/15226514.2019.1658706, PMID: 31475567

[ref18] Chepurnov V. A. Mann D. G. Von Dassow P. Vanormelingen P. Gillard J. Inze D.. (2008). In search of new tractable diatoms for experimental biology. BioEssays 30, 692–702. doi: 10.1002/bies.20773, PMID: 18536039

[ref19] Coppens J. Lindeboom R. Muys M. Coessens W. Alloul A. Meerbergen K.. (2016). Nitrification and microalgae cultivation for two-stage biological nutrient valorization from source separated urine. Bioresour. Technol. 211, 41–50. doi: 10.1016/j.biortech.2016.03.001, PMID: 26998796

[ref20] Cuaresma M. Janssen M. Vilchez C. Wijffels R. H. (2009). Productivity of Chlorella sorokiniana in a short light-path (SLP) panel photobioreactor under high irradiance. Biotechnol. Bioeng. 104, 352–359. doi: 10.1002/bit.22394, PMID: 19517522

[ref21] Danesi E. D. G. Rangel-Yagui D. O. De Carvalho J. C. M. Sato S. (2002). An investigation of effect of replacing nitrate by urea in the growth and production of chlorophyll by Spirulina platensis. Biomass Bioenergy 23, 261–269. doi: 10.1016/S0961-9534(02)00054-5

[ref002] Darienko T. Rad-Menéndez C. Campbell C. Pröschold T. (2019). Are there any true marine Chlorella species? Molecular phylogenetic assessment and ecology of marine Chlorella-like organisms, including a description of Droopiella gen. nov. Systematics and biodiversity 17, 811–829. doi: 10.1080/14772000.2019.1690597 32256217 PMC7077435

[ref22] de Wilt A. Butkovskyi A. Tuantet K. Leal L. H. Fernandes T. V. Langenhoff A.. (2016). Micropollutant removal in an algal treatment system fed with source separated wastewater streams. J. Hazard. Mater. 304, 84–92. doi: 10.1016/j.jhazmat.2015.10.033, PMID: 26546707

[ref23] Detrell G. Helisch H. Keppler J. Martin J. Henn N. (2020). “Microalgae for combined air revitalization and biomass production for space applications,” in *From Biofiltration to Promising Options in Gaseous Fluxes Biotreatment*. eds. Gabriela S. Éric D. (Elsevier Inc), 419–445.

[ref24] Dismukes G. C. Carrieri D. Bennette N. Ananyev G. M. Posewitz M. C. (2008). Aquatic phototrophs: efficient alternatives to land-based crops for biofuels. Curr. Opin. Biotechnol. 19, 235–240. doi: 10.1016/j.copbio.2008.05.007, PMID: 18539450

[ref25] Feng D. L. Wu Z. C. (2006). Culture of Spirulina platensis in human urine for biomass production and O(2) evolution. J Zhejiang Univ Sci B 7, 34–37. doi: 10.1631/jzus.2006.B0034, PMID: 16365923 PMC1361757

[ref26] Gao F. Li C. Yang Z.-H. Zeng G.-M. Feng L.-J. Liu J.-Z.. (2016). Continuous microalgae cultivation in aquaculture wastewater by a membrane photobioreactor for biomass production and nutrients removal. Ecol. Eng. 92, 55–61. doi: 10.1016/j.ecoleng.2016.03.046

[ref27] Gao F. Peng Y.-Y. Li C. Cui W. Yang Z.-H. Zeng G.-M. (2018). Coupled nutrient removal from secondary effluent and algal biomass production in membrane photobioreactor (MPBR): effect of HRT and long-term operation. Chem. Eng. J. 335, 169–175. doi: 10.1016/j.cej.2017.10.151

[ref28] Gòdia F. Albiol J. Montesinos J. L. Pérez J. Creus N. Cabello F.. (2002). MELISSA: a loop of interconnected bioreactors to develop life support in space. J. Biotechnol. 99, 319–330. doi: 10.1016/S0168-1656(02)00222-5, PMID: 12385718

[ref29] Golder D. Rana S. Sarkar D. Jana B. B. (2007). Human urine is an excellent liquid waste for the culture of fish food organism, Moina micrura. Ecol. Eng. 30, 326–332. doi: 10.1016/j.ecoleng.2007.04.002

[ref30] Gutierrez-Salmean G. Fabila-Castillo L. Chamorro-Cevallos G. (2015). Nutritional and toxicological aspects of spirulina (Arthrospira). Nutr. Hosp. 32, 34–40. doi: 10.3305/nh.2015.32.1.9001, PMID: 26262693

[ref31] He C. Wang J. Fu L. Zhao C. Huo J. (2022). Associative vs. dissociative mechanism: Electrocatalysis of nitric oxide to ammonia. Chin. Chem. Lett. 33, 1051–1057. doi: 10.1016/j.cclet.2021.09.009

[ref32] Holdmann C. Schmid-Staiger U. Hornstein H. Hirth T. (2018). Keeping the light energy constant—cultivation of Chlorella sorokiniana at different specific light availabilities and different photoperiods. Algal Res. 29, 61–70. doi: 10.1016/j.algal.2017.11.005

[ref33] Hulatt C. J. Lakaniemi A.-M. Puhakka J. A. Thomas D. N. (2012). Energy demands of nitrogen supply in mass cultivation of two commercially important microalgal species, Chlorella vulgaris and *Dunaliella tertiolecta*. Bioenergy Res. 5, 669–684. doi: 10.1007/s12155-011-9175-x

[ref34] Hydrobiology, F.A.C.C.O.T.I.O. (2013). *Spirulina platensis* [Online]. Available at: http://algae.ihb.ac.cn/english/algaeDetail.aspx?id=448 (Accessed October 4, 2022).

[ref35] Jaatinen S. Lakaniemi A. M. Rintala J. (2016). Use of diluted urine for cultivation of Chlorella vulgaris. Environ. Technol. 37, 1159–1170. doi: 10.1080/09593330.2015.1105300, PMID: 26508358

[ref36] Jelic A. Rodriguez-Mozaz S. Barcelo D. Gutierrez O. (2015). Impact of in-sewer transformation on 43 pharmaceuticals in a pressurized sewer under anaerobic conditions. Water Res. 68, 98–108. doi: 10.1016/j.watres.2014.09.033, PMID: 25462720

[ref37] Källqvist T. Svenson A. (2003). Assessment of ammonia toxicity in tests with the microalga, Nephroselmis pyriformis, Chlorophyta. Water Res. 37, 477–484. doi: 10.1016/S0043-1354(02)00361-5, PMID: 12688681

[ref38] Karlson B. Andreasson A. Johansen M. Karlberg M. Loo A. Skjevik A.-T., (2020). *Nordic Microalgae [online]*. World-Wide Electronic Publication. Available at: http://nordicmicroalgae.org (Accessed October 10 2022).

[ref39] Kim J.-Y. Kim H.-W. (2017). Photoautotrophic microalgae screening for tertiary treatment of livestock wastewater and bioresource recovery. Water 9:192. doi: 10.3390/w9030192

[ref40] Kim G. Y. Yun Y. M. Shin H. S. Kim H. S. Han J. I. (2015). Scenedesmus-based treatment of nitrogen and phosphorus from effluent of anaerobic digester and bio-oil production. Bioresour. Technol. 196, 235–240. doi: 10.1016/j.biortech.2015.07.091, PMID: 26247974

[ref41] Kinnunen V. Rintala J. (2016). The effect of low-temperature pretreatment on the solubilization and biomethane potential of microalgae biomass grown in synthetic and wastewater media. Bioresour. Technol. 221, 78–84. doi: 10.1016/j.biortech.2016.09.017, PMID: 27639227

[ref42] Lakaniemi A. M. Intihar V. M. Tuovinen O. H. Puhakka J. A. (2012). Growth of Chlorella vulgaris and associated bacteria in photobioreactors. Microb. Biotechnol. 5, 69–78. doi: 10.1111/j.1751-7915.2011.00298.x, PMID: 21936882 PMC3815273

[ref43] Larsen T. A. Riechmann M. E. Udert K. M. (2021). State of the art of urine treatment technologies: a critical review. Water Res. X 13:100114. doi: 10.1016/j.wroa.2021.100114, PMID: 34693239 PMC8517923

[ref44] Li J. Ou R. Liao H. Ma J. Sun L. Jin Q.. (2022). Natural lighting enhancing the algae proliferation and nitrogen removal in membrane-aerated bacterial-algal biofilm reactor. Sci. Total Environ. 851:158063. doi: 10.1016/j.scitotenv.2022.158063, PMID: 35981577

[ref45] Lienert J. Burki T. Escher B. I. (2007). Reducing micropollutants with source control: substance flow analysis of 212 pharmaceuticals in faeces and urine. Water Sci. Technol. 56, 87–96. doi: 10.2166/wst.2007.560, PMID: 17881841

[ref46] Liu Z.-Y. Wang G.-C. Zhou B.-C. (2008). Effect of iron on growth and lipid accumulation in Chlorella vulgaris. Bioresour. Technol. 99, 4717–4722. doi: 10.1016/j.biortech.2007.09.073, PMID: 17993270

[ref47] Liu Z. Zhao Q. Wang K. Lee D. Qiu W. Wang J. (2008). Urea hydrolysis and recovery of nitrogen and phosphorous as MAP from stale human urine. J. Environ. Sci. 20, 1018–1024. doi: 10.1016/S1001-0742(08)62202-0, PMID: 18817084

[ref48] Lu F. Ji J. Shao L. He P. (2013). Bacterial bioaugmentation for improving methane and hydrogen production from microalgae. Biotechnol. Biofuels 6:92. doi: 10.1186/1754-6834-6-92, PMID: 23815806 PMC3699423

[ref49] Luo Y. Le-Clech P. Henderson R. K. (2017). Simultaneous microalgae cultivation and wastewater treatment in submerged membrane photobioreactors: a review. Algal Res. 24, 425–437. doi: 10.1016/j.algal.2016.10.026

[ref50] Ma J. Dai R. Chen M. Khan S. J. Wang Z. (2018a). Applications of membrane bioreactors for water reclamation: micropollutant removal, mechanisms and perspectives. Bioresour. Technol. 269, 532–543. doi: 10.1016/j.biortech.2018.08.121, PMID: 30195697

[ref51] Ma J. He C. He D. Zhang C. Waite T. D. (2018b). Analysis of capacitive and electrodialytic contributions to water desalination by flow-electrode CDI. Water Res. 144, 296–303. doi: 10.1016/j.watres.2018.07.049, PMID: 30053621

[ref52] Ma J. Wang Z. He D. Li Y. Wu Z. (2015). Long-term investigation of a novel electrochemical membrane bioreactor for low-strength municipal wastewater treatment. Water Res. 78, 98–110. doi: 10.1016/j.watres.2015.03.033, PMID: 25917391

[ref53] Ma J. Wang Z. Yang Y. Mei X. Wu Z. (2013a). Correlating microbial community structure and composition with aeration intensity in submerged membrane bioreactors by 454 high-throughput pyrosequencing. Water Res. 47, 859–869. doi: 10.1016/j.watres.2012.11.013, PMID: 23200801

[ref54] Ma J. Wang Z. Zhang J. Waite T. D. Wu Z. (2017). Cost-effective chlorella biomass production from dilute wastewater using a novel photosynthetic microbial fuel cell (PMFC). Water Res. 108, 356–364. doi: 10.1016/j.watres.2016.11.016, PMID: 27836177

[ref55] Ma J. Wang Z. Zou X. Feng J. Wu Z. (2013b). Microbial communities in an anaerobic dynamic membrane bioreactor (AnDMBR) for municipal wastewater treatment: comparison of bulk sludge and cake layer. Process Biochem. 48, 510–516. doi: 10.1016/j.procbio.2013.02.003

[ref56] Maurer M. Schwegler P. Larsen T. A. (2003). Nutrients in urine: energetic aspects of removal and recovery. Water Sci. Technol. 48, 37–46. doi: 10.2166/wst.2003.0011, PMID: 12926619

[ref57] Monetti J. Nieradzik L. Freguia S. Choi P. M. O’brien J. W. Thomas K. V.. (2022). Urea hydrolysis and long-term storage of source-separated urine for reuse as fertiliser is insufficient for the removal of anthropogenic micropollutants. Water Res. 222:118891. doi: 10.1016/j.watres.2022.118891, PMID: 35907300

[ref58] Morais Junior W. G. Gorgich M. Corrêa P. S. Martins A. A. Mata T. M. Caetano N. S. (2020). Microalgae for biotechnological applications: cultivation, harvesting and biomass processing. Aquaculture 528:735562. doi: 10.1016/j.aquaculture.2020.735562

[ref59] Munoz C. F. Sudfeld C. Naduthodi M. I. S. Weusthuis R. A. Barbosa M. J. Wijffels R. H.. (2021). Genetic engineering of microalgae for enhanced lipid production. Biotechnol. Adv. 52:107836. doi: 10.1016/j.biotechadv.2021.107836, PMID: 34534633

[ref60] Muys M. Coppens J. Boon N. Vlaeminck S. E. (2018). Photosynthetic oxygenation for urine nitrification. Water Sci. Technol. 78, 183–194. doi: 10.2166/wst.2018.200, PMID: 30101801

[ref61] Nazari S. Zinatizadeh A. A. Mirghorayshi M. Van Loosdrecht M. C. M. (2020). Waste or gold? Bioelectrochemical resource recovery in source-separated urine. Trends Biotechnol. 38, 990–1006. doi: 10.1016/j.tibtech.2020.03.007 32345461

[ref62] Nguyen T.-T. Bui X.-T. Ngo H. H. Nguyen T.-T.-D. Nguyen K.-Q. Nguyen H.-H.. (2021). Nutrient recovery and microalgae biomass production from urine by membrane photobioreactor at low biomass retention times. Sci. Total Environ. 785:147423. doi: 10.1016/j.scitotenv.2021.147423

[ref63] Nguyen T.-T. Lee C. Field R. W. Kim I. S. (2020a). Insight into organic fouling behavior in polyamide thin-film composite forward osmosis membrane: critical flux and its impact on the economics of water reclamation. J. Membr. Sci. 606:118118. doi: 10.1016/j.memsci.2020.118118

[ref65] Nguyen T. T. Nguyen T. T. An Binh Q. Bui X. T. Ngo H. H. Vo H. N. P.. (2020b). Co-culture of microalgae-activated sludge for wastewater treatment and biomass production: exploring their role under different inoculation ratios. Bioresour. Technol. 314:123754. doi: 10.1016/j.biortech.2020.123754, PMID: 32650264

[ref66] Organisms C. C. O. A. (2020). Chlorella sorokiniana [Online]. Culture Collection of Autotrophic Organisms. Available at: https://ccala.butbn.cas.cz/en/chlorella-sorokiniana-shihira-et-krauss (Accessed October 4, 2022).

[ref67] Pawar S. B. (2016). Process engineering aspects of vertical column Photobioreactors for mass production of microalgae. ChemBioEng Reviews 3, 101–115. doi: 10.1002/cben.201600003

[ref68] Piltz B. Melkonian M. (2017). Immobilized microalgae for nutrient recovery from source-separated human urine. J. Appl. Phycol. 30, 421–429. doi: 10.1007/s10811-017-1266-4

[ref69] Pires J. C. M. Alvim-Ferraz M. C. M. Martins F. G. Simões M. (2012). Carbon dioxide capture from flue gases using microalgae: engineering aspects and biorefinery concept. Renew. Sust. Energ. Rev. 16, 3043–3053. doi: 10.1016/j.rser.2012.02.055

[ref70] Porra R. J. Thompson W. A. Kriedemann P. E. (1989). Determination of accurate extinction coefficients and simultaneous equations for assaying chlorophylls a and b extracted with four different solvents: verification of the concentration of chlorophyll standards by atomic absorption spectroscopy. Biochimica et Biophysica Acta (BBA) – Bioenergetics 975, 384–394. doi: 10.1016/S0005-2728(89)80347-0

[ref71] Posadas E. Morales M. D. M. Gomez C. Acién F. G. Muñoz R. (2015). Influence of pH and CO2 source on the performance of microalgae-based secondary domestic wastewater treatment in outdoors pilot raceways. Chem. Eng. J. 265, 239–248. doi: 10.1016/j.cej.2014.12.059

[ref72] Praveen P. Xiao W. Lamba B. Loh K.-C. (2019). Low-retention operation to enhance biomass productivity in an algal membrane photobioreactor. Algal Res. 40:101487. doi: 10.1016/j.algal.2019.101487

[ref73] Quinn B. Gagné F. Blaise C. (2009). Evaluation of the acute, chronic and teratogenic effects of a mixture of eleven pharmaceuticals on the cnidarian, Hydra attenuata. Sci. Total Environ. 407, 1072–1079. doi: 10.1016/j.scitotenv.2008.10.022, PMID: 19013635

[ref74] Raeisossadati M. Moheimani N. R. Parlevliet D. (2020). Red luminescent solar concentrators to enhance Scenedesmus sp. biomass productivity. Algal Res. 45:101771. doi: 10.1016/j.algal.2019.101771

[ref75] Remmers I. M. Wijffels R. H. Barbosa M. J. Lamers P. P. (2018). Can we approach theoretical lipid yields in microalgae? Trends Biotechnol. 36, 265–276. doi: 10.1016/j.tibtech.2017.10.020, PMID: 29395347

[ref76] Ren L. Chen M. Ma J. Li Y. Wang Z. (2022a). Pd–O_2_ interaction and singlet oxygen formation in a novel reactive electrochemical membrane for ultrafast sulfamethoxazole oxidation. Chem. Eng. J. 428:131194. doi: 10.1016/j.cej.2021.131194

[ref77] Ren L. Ma J. Chen M. Qiao Y. Dai R. Li X.. (2022b). Recent advances in electrocatalytic membrane for the removal of micropollutants from water and wastewater. iScience 25:104342. doi: 10.1016/j.isci.2022.104342, PMID: 35602955 PMC9117875

[ref78] Revellame E. D. Aguda R. Chistoserdov A. Fortela D. L. Hernandez R. A. Zappi M. E. (2021). Microalgae cultivation for space exploration: assessing the potential for a new generation of waste to human life-support system for long duration space travel and planetary human habitation. Algal Res. 55:102258. doi: 10.1016/j.algal.2021.102258

[ref79] Rezvani S. Saadaoui I. Al Jabri H. Moheimani N. R. (2022). Techno-economic modelling of high-value metabolites and secondary products from microalgae cultivated in closed photobioreactors with supplementary lighting. Algal Res. 65:102733. doi: 10.1016/j.algal.2022.102733

[ref80] Rodushkin I. Ödman F. (2001). Application of inductively coupled plasma sector field mass spectrometry for elemental analysis of urine. J. Trace Elem. Med. Biol. 14, 241–247. doi: 10.1016/S0946-672X(01)80010-9, PMID: 11396785

[ref81] Schuler J. F. Diller V. M. Kersten H. J. (1953). Preferential assimilation of ammonium ion by Chlorella vulgaris. Plant Physiol. 28, 299–303. doi: 10.1104/pp.28.2.299, PMID: 16654542 PMC540382

[ref82] Singh P. Guldhe A. Kumari S. Rawat I. Bux F. (2015). Investigation of combined effect of nitrogen, phosphorus and iron on lipid productivity of microalgae Ankistrodesmus falcatus KJ671624 using response surface methodology. Biochem. Eng. J. 94, 22–29. doi: 10.1016/j.bej.2014.10.019

[ref83] Soares F. R. Martins G. Seo E. S. (2013). An assessment of the economic aspects of CO2 sequestration in a route for biodiesel production from microalgae. Environ. Technol. 34, 1777–1781. doi: 10.1080/09593330.2013.816784 24350434

[ref84] Song J. Wang X. Ma J. Wang X. Wang J. Xia S.. (2018). Removal of *Microcystis aeruginosa* and microcystin-LR using a graphitic-C3N4/TiO2 floating photocatalyst under visible light irradiation. Chem. Eng. J. 348, 380–388. doi: 10.1016/j.cej.2018.04.182

[ref85] Sun L. Ma J. Li L. Tian Y. Zhang Z. Liao H.. (2020). Exploring the essential factors of performance improvement in sludge membrane bioreactor technology coupled with symbiotic algae. Water Res. 181:115843. doi: 10.1016/j.watres.2020.115843, PMID: 32422450

[ref86] Sun H. Zhao W. Mao X. Li Y. Wu T. Chen F. (2018). High-value biomass from microalgae production platforms: strategies and progress based on carbon metabolism and energy conversion. Biotechnol. Biofuels 11:227. doi: 10.1186/s13068-018-1225-6, PMID: 30151055 PMC6100726

[ref87] Sydney E. B. Sturm W. De Carvalho J. C. Thomaz-Soccol V. Larroche C. Pandey A.. (2010). Potential carbon dioxide fixation by industrially important microalgae. Bioresour. Technol. 101, 5892–5896. doi: 10.1016/j.biortech.2010.02.088, PMID: 20350804

[ref88] Tao R. Kinnunen V. Praveenkumar R. Lakaniemi A. M. Rintala J. A. (2017a). Comparison of *Scenedesmus acuminatus* and Chlorella vulgaris cultivation in liquid digestates from anaerobic digestion of pulp and paper industry and municipal wastewater treatment sludge. J. Appl. Phycol. 29, 2845–2856. doi: 10.1007/s10811-017-1175-6

[ref89] Tao R. Lakaniemi A. M. Rintala J. A. (2017b). Cultivation of Scenedesmus acuminatus in different liquid digestates from anaerobic digestion of pulp and paper industry biosludge. Bioresour. Technol. 245, 706–713. doi: 10.1016/j.biortech.2017.08.218, PMID: 28917106

[ref90] Tuantet K. (2015). Microalgae Cultivation for Nutrient Recovery from Human Urine. PhD. Wageningen, NL: Wageningen University.

[ref91] Tuantet K. Janssen M. Temmink H. Zeeman G. Wijffels R. H. Buisman C. J. N. (2013). Microalgae growth on concentrated human urine. J. Appl. Phycol. 26, 287–297. doi: 10.1007/s10811-013-0108-2

[ref92] Tuantet K. Temmink H. Zeeman G. Janssen M. Wijffels R. H. Buisman C. J. (2014). Nutrient removal and microalgal biomass production on urine in a short light-path photobioreactor. Water Res. 55, 162–174. doi: 10.1016/j.watres.2014.02.027, PMID: 24607312

[ref93] Tuantet K. Temmink H. Zeeman G. Wijffels R. H. Buisman C. J. N. Janssen M. (2019). Optimization of algae production on urine. Algal Res. 44:101667. doi: 10.1016/j.algal.2019.101667 24607312

[ref94] Udert K. M. Buckley C. A. Wächter M. Mcardell C. S. Kohn T. Strande L.. (2016). Technologies for the treatment of source-separated urine in the eThekwini municipality. Water SA 41, 2571–2582. doi: 10.4314/wsa.v41i2.06

[ref95] Udert K. M. Larsen T. A. Biebow M. Gujer W. (2003). Urea hydrolysis and precipitation dynamics in a urine-collecting system. Water Res. 37, 2571–2582. doi: 10.1016/S0043-1354(03)00065-4, PMID: 12753834

[ref96] Unpaprom Y. Tipnee S. Rameshprabu R. (2015). Biodiesel from green alga *Scenedesmus acuminatus*. Int. J. Sustainable Green Energy 4, 1–6. doi: 10.11648/j.ijrse.s.2015040101.11

[ref97] Verstraete W. Tanghe T. Janssens I. (1997). Micropollutants: a bottleneck in sustainable wastewater treatment. Water Sci. Technol. 35, 13–26. doi: 10.2166/wst.1997.0349

[ref98] Vílchez C. Vega J. (1995). Nitrite uptake by immobilized *Chlamydomonas reinhardtii* cells growing in airlift reactors. Enzym. Microb. Technol. 17, 386–390. doi: 10.1016/0141-0229(94)00037-R

[ref99] Wang H. Wang X. Ma J. Xia P. Zhao J. (2017). Removal of cadmium (II) from aqueous solution: a comparative study of raw attapulgite clay and a reusable waste–struvite/attapulgite obtained from nutrient-rich wastewater. J. Hazard. Mater. 329, 66–76. doi: 10.1016/j.jhazmat.2017.01.025, PMID: 28135656

[ref100] Wang Z. Yu H. Ma J. Zheng X. Wu Z. (2013). Recent advances in membrane bio-technologies for sludge reduction and treatment. Biotechnol. Adv. 31, 1187–1199. doi: 10.1016/j.biotechadv.2013.02.004, PMID: 23466365

[ref101] Wang J. Zhou W. Chen H. Zhan J. He C. Wang Q. (2019). Ammonium nitrogen tolerant chlorella strain screening and its damaging effects on photosynthesis. Front. Microbiol. 9:3250. doi: 10.3389/fmicb.2018.03250 30666245 PMC6330332

[ref102] Wilsenach J. A. Schuurbiers C. A. Van Loosdrecht M. C. (2007). Phosphate and potassium recovery from source separated urine through struvite precipitation. Water Res. 41, 458–466. doi: 10.1016/j.watres.2006.10.014, PMID: 17126877

[ref103] Wilsenach J. A. Van Loosdrecht M. C. (2006). Integration of processes to treat wastewater and source-separated urine. J. Environ. Eng. 132, 331–341. doi: 10.1061/(ASCE)0733-9372(2006)132:3(331)

[ref104] Yang K. Lin H. Feng X. Jiang J. Ma J. Yang Z. (2022). Energy-efficient removal of trace antibiotics from low-conductivity water using a Ti4O7 reactive electrochemical ceramic membrane: matrix effects and implications for byproduct formation. Water Res. 224:119047. doi: 10.1016/j.watres.2022.119047, PMID: 36103779

[ref105] Yang C. Liu H. Li M. Yu C. Yu G. (2008). Treating urine by Spirulina platensis. Acta Astronaut. 63, 1049–1054. doi: 10.1016/j.actaastro.2008.03.008

[ref106] Yang J. Xu M. Zhang X. Hu Q. Sommerfeld M. Chen Y. (2011). Life-cycle analysis on biodiesel production from microalgae: water footprint and nutrients balance. Bioresour. Technol. 102, 159–165. doi: 10.1016/j.biortech.2010.07.017, PMID: 20675125

[ref107] Yin C. Aroua M. Daud W. (2007). Review of modifications of activated carbon for enhancing contaminant uptakes from aqueous solutions. Sep. Purif. Technol. 52, 403–415. doi: 10.1016/j.seppur.2006.06.009

[ref108] Yuan D. Liu J. Wang H. Hu Q. Gong Y. (2022). Biodiversity and seasonal variation of microzooplankton contaminating pilot-scale cultures of Chlorella sorokiniana. Algal Res. 64:102722. doi: 10.1016/j.algal.2022.102722

[ref109] Zhang J. Giannis A. Chang V. W. Ng B. J. Wang J. Y. (2013). Adaptation of urine source separation in tropical cities: process optimization and odor mitigation. J. Air Waste Manag. Assoc. 63, 472–481. doi: 10.1080/10962247.2013.763306, PMID: 23687732

[ref110] Zhang S. Lim C. Y. Chen C. L. Liu H. Wang J. Y. (2014). Urban nutrient recovery from fresh human urine through cultivation of Chlorella sorokiniana. J. Environ. Manag. 145, 129–136. doi: 10.1016/j.jenvman.2014.06.013, PMID: 25016102

[ref111] Zhang C. Ma J. He D. Waite T. D. (2018). Capacitive membrane stripping for ammonia recovery (CapAmm) from dilute wastewaters. Environ. Sci. Technol. Lett. 5, 43–49. doi: 10.1021/acs.estlett.7b00534 30458615

[ref112] Zhang D. Yang S. Fang X. Li H. Chen X. Yan D. (2022). In situ localization of BiVO4 onto two-dimensional MXene promoting photoelectrochemical nitrogen reduction to ammonia. Chin. Chem. Lett. 33, 4669–4674. doi: 10.1016/j.cclet.2022.02.001

[ref113] Zhu L. Wang Z. Shu Q. Takala J. Hiltunen E. Feng P.. (2013). Nutrient removal and biodiesel production by integration of freshwater algae cultivation with piggery wastewater treatment. Water Res. 47, 4294–4302. doi: 10.1016/j.watres.2013.05.004, PMID: 23764580

[ref114] Zittelli G. C. Rodolfi L. Bassi N. Biondi N. Tredici M. R. (2013). “Photobioreactors for microalgal biofuel production,” in *Algae for Biofuels and Energy*. eds. Michael A. B. Navid R. M. (Springer Dordrecht), 115–131.

